# The strength in numbers: comprehensive characterization of house dust using complementary mass spectrometric techniques

**DOI:** 10.1007/s00216-019-01615-6

**Published:** 2019-03-04

**Authors:** Pawel Rostkowski, Peter Haglund, Reza Aalizadeh, Nikiforos Alygizakis, Nikolaos Thomaidis, Joaquin Beltran Arandes, Pernilla Bohlin Nizzetto, Petra Booij, Hélène Budzinski, Pamela Brunswick, Adrian Covaci, Christine Gallampois, Sylvia Grosse, Ralph Hindle, Ildiko Ipolyi, Karl Jobst, Sarit L. Kaserzon, Pim Leonards, Francois Lestremau, Thomas Letzel, Jörgen Magnér, Hidenori Matsukami, Christoph Moschet, Peter Oswald, Merle Plassmann, Jaroslav Slobodnik, Chun Yang

**Affiliations:** 1 NILU—Norwegian Institute for Air Research, 2027 Kjeller, Norway; 2 Umeå University, 90187 Umeå, Sweden; 3 Department of Chemistry, University of Athens, 157 71 Athens, Greece; 4 Environmental Institute, 972 41 Kos, Slovak Republic; 5 Research Institute for Pesticides and Water, University Jaume I, 12071 Castelló, Spain; 6 Research Centre for Toxic Compounds in the Environment, 611 37 Brno, Czech Republic; 7 University of Bordeaux, 33405 Talence Cedex, France; 8 Environment and Climate Change Canada, North Vancouver, V7H 1B1 Canada; 9 Toxicological Center, University of Antwerp, 2610 Wilrijk, Belgium; 10 Technical University of Munich, 85748 Garching, Germany; 11 Vogon Laboratory Services Ltd, Cochrane, AB T4C 0A3 Canada; 12 Ontario Ministry of Environment and Climate Change, Etobicoke, ON M9P 3V6 Canada; 13 Queensland Alliance for Environmental Health Sciences (QAEHS), University of Queensland, Woolloongabba, QLD 4102 Australia; 14 VU University Amsterdam, 1081 HV Amsterdam, The Netherlands; 15 INERIS, Parc Technologique ALATA, 60550 Verneuil-en-Halatte, France; 16 IVL Swedish Environmental Research Institute, 114 27 Stockholm, Sweden; 17 Present Address: Swedish Chemicals Agency (KemI), 172 67 Sundbyberg, Sweden; 18 National Institute for Environmental Studies, Tsukuba, 305-8506 Japan; 19 University of California, Davis, CA 95616 USA; 20 Department of Environmental Science and Analytical Chemistry (ACES), Stockholm University, 106 91 Stockholm, Sweden; 21 Environment and Climate Change Canada, Ottawa, ON K1V 1C7 Canada

**Keywords:** House dust, Suspect and nontarget analysis, Collaborative trial, Complementary analytical techniques, Mass spectrometry

## Abstract

Untargeted analysis of a composite house dust sample has been performed as part of a collaborative effort to evaluate the progress in the field of suspect and nontarget screening and build an extensive database of organic indoor environment contaminants. Twenty-one participants reported results that were curated by the organizers of the collaborative trial. In total, nearly 2350 compounds were identified (18%) or tentatively identified (25% at confidence level 2 and 58% at confidence level 3), making the collaborative trial a success. However, a relatively small share (37%) of all compounds were reported by more than one participant, which shows that there is plenty of room for improvement in the field of suspect and nontarget screening. An even a smaller share (5%) of the total number of compounds were detected using both liquid chromatography–mass spectrometry (LC-MS) and gas chromatography–mass spectrometry (GC-MS). Thus, the two MS techniques are highly complementary. Most of the compounds were detected using LC with electrospray ionization (ESI) MS and comprehensive 2D GC (GC×GC) with atmospheric pressure chemical ionization (APCI) and electron ionization (EI), respectively. Collectively, the three techniques accounted for more than 75% of the reported compounds. Glycols, pharmaceuticals, pesticides, and various biogenic compounds dominated among the compounds reported by LC-MS participants, while hydrocarbons, hydrocarbon derivatives, and chlorinated paraffins and chlorinated biphenyls were primarily reported by GC-MS participants. Plastics additives, flavor and fragrances, and personal care products were reported by both LC-MS and GC-MS participants. It was concluded that the use of multiple analytical techniques was required for a comprehensive characterization of house dust contaminants. Further, several recommendations are given for improved suspect and nontarget screening of house dust and other indoor environment samples, including the use of open-source data processing tools. One of the tools allowed provisional identification of almost 500 compounds that had not been reported by participants.

**Electronic supplementary material:**

The online version of this article (10.1007/s00216-019-01615-6) contains supplementary material, which is available to authorized users.

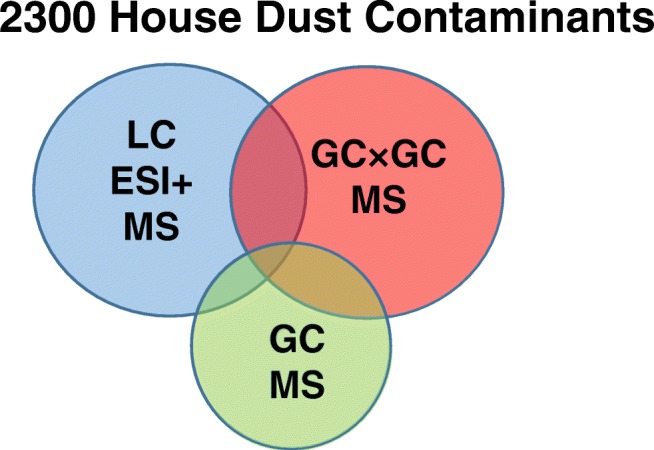

## Introduction

The indoor environment is increasingly gaining attention as an important source of human exposure to environmental contaminants including pesticides, polybrominated diphenyl ethers (PBDEs), polycyclic aromatic hydrocarbons (PAHs), plasticizers, organophosphorus flame retardants, bisphenols, parabens, and other chemicals of concern for human health [1–7]. Pollution in the indoor environment is believed to contribute to a range of adverse effects, including respiratory diseases, cancer, and neuropsychological disorders [8–10]. Although dust is one of the most frequently studied matrices in the indoor environment, due to its complexity, analysis is almost exclusively limited to targeted analyses. Rapid development of new building materials, furnishings, and consumer products and lower air exchange rates for improved energy efficiency can be the factors increasing the accumulation of contaminants in indoor environments. It is anticipated that household dust should be further examined for the presence of other chemicals of human health concern to provide a more complete understanding of chemical exposure indoors. House dust is considered as an important exposure medium, in particular for infants and toddlers, who are at highest risk owing to hand-to-mouth activities. It is thought that the ingestion of settled dust may constitute a significant part of the exposure to some phthalates [11], polybrominated diphenyl ethers [12], and pesticides [13]. Hence, settled dust could be a global indicator of residential contamination, in particular for semivolatile and nonvolatile contaminants, and studies of household dust can be considered as an early warning system of environmental contamination. The screening of contaminants in dust facilitates the identification of new environmental hazards much earlier than through screening of ambient (outdoor) environmental matrices, where dispersion tends to reduce contaminant concentrations. Searching for unknown and even unanticipated chemicals requires a nontargeted analytical approach. So far, the field of suspect and nontarget screening is rapidly expanding, with examples of successful application, e.g., for emerging contaminants in water samples [14–18], but its application to the indoor environment was rather limited with some successful applications reported only recently [19–22].

In response to the growing interest in further development and harmonization of suspect and nontarget screening approaches and their application to the indoor environment, the NORMAN network (Network of reference laboratories, research centres and related organisations for monitoring of emerging environmental substances) organized a collaborative nontarget screening trial on composite household dust. Each participating organization was requested to analyze the test sample using established mass spectrometry (MS) techniques in their laboratory, declare a number of substances present in the sample, and perform provisional identification using target, suspect, and nontarget screening approaches. Together with the test sample, mixtures of analytical standards suitable for liquid chromatography (LC) and gas chromatography (GC), respectively, were provided for the calculation of retention time index (RTI) and retention index (RI) information. RIs have a significant role in nontarget screening as they can be used to support or reject a candidate structure. In future studies, suspect lists with associated RIs will have a great value, e.g., in retrospective screening of compounds of emerging concern using data stored in digital archives.

The study aimed to (a) evaluate progress in the field of suspect and nontarget screening of dust, (b) build an extensive database of semivolatile and nonvolatile organic contaminants that could be applied in future screening of the indoor environment, (c) assess the degree of complementarity of instrumental analytical techniques, and (d) present recommendations for successful suspect and nontarget screening. To our knowledge, this is the first collaborative effort on untargeted analysis of indoor dust.

## Materials and methods

### Description of the samples and participation in the trial

All dust samples were obtained from a larger, homogenized dust sample, which was a composite of residential dust obtained from household vacuum bags collected from homes around Toronto, Canada, in 2015. The dust was preprocessed by sieving with a coarse 1-mm sieve to aid in homogenization and stored at − 18 °C. The dust sample was collected to be used in another interlaboratory study aimed at halogenated flame-retardant contaminants in indoor dust and has been checked for homogeneity. Aliquots of 250 mg of the dust were transferred to brown glass vials that were dispatched in January 2016 with standard mixtures for use in the calculation of retention index information: alkane standards for GC-MS techniques and 10 substances for LC-MS techniques (Electronic supplementary material (ESM) Table S1a). In addition, participants were later requested to analyze two additional standard mixtures, for negative electrospray ionization (ESI) and positive ESI, respectively, each of them containing 18 substances (ESM Table S1b) to facilitate quantitative structure–retention relationship (QSRR)-based prediction of retention times of unknown compounds.

All participants were requested to measure these standards and report the results by June 2016. The reporting was done using data collection templates that included details related to the chromatographic and mass spectrometric methods and related to the reported compounds, e.g., retention time (RT), *m*/*z*, intensity, intensity of blank, MS/MS data, type of workflow, proposed ID, molecular formula, CAS, and identification confidence level. Twenty-seven participants from 26 organizations representing 15 countries registered to participate in the study. Seventeen participants registered for LC-MS and GC-MS techniques, three only for the GC-MS, and seven for LC-MS only. Out of these, 20 datasets were received for the LC-MS techniques and 14 for the GC-MS techniques. One participant officially withdrew from the trial for both techniques and one for the GC-MS technique only. The participants’ list included institutions with various levels of experience in performing suspect and nontarget methods (i.e., some were performing nontarget analysis for the first time, while others were more experienced). Table 1 shows a summary of the contributions received.

**Table 1:** Contributing laboratories (coded) and their geographic distribution, summary statistics on the number of data and tentatively identified compounds, and type of workflows used (self-reported)

Code	Region	GC-MS or LC-MS	Total number of compounds	Number of compounds		Self-reported workflow		
				LC-MS	GC-MS	Target (%)	Suspect (%)	Nontarget (%)
Lab 1	Asia-Pacific, N. America	Both	591	457	134	6	5	89
Lab 2	Europe	Both	583	49	534	2	6	92
Lab 3	Asia-Pacific, N. America	GC-MS	525	–	525	0	0	100
Lab 4	Asia-Pacific, N. America	Both	417	271	146	15	14	78
Lab 5	Europe	Both	415	293	122	0	2	98
Lab 6	Europe	LC-MS	337	337	–	25	75	0
Lab 7	Europe	Both	287	57	230	1	11	88
Lab 8	Asia-Pacific, N. America	Both	216	25	191	29	19	52
Lab 9	Europe	Both	211	28	183	15	11	74
Lab 10	Europe	Both	122	77	45	19	26	55
Lab 11	Asia-Pacific, N. America	LC-MS	121	121	–	0	0	100
Lab 12	Europe	LC-MS	186	186	–	3	0	97
Lab 13	Europe	Both	180	143	37	0	0	100
Lab 14	Europe	LC-MS	77	77	–	0	58	42
Lab 15	Europe	LC-MS	69	69	–	38	48	14
Lab 16	Europe	Both	68	21	47	22	1	76
Lab 17	Europe	Both	57	46	11	91	0	9
Lab 18	Asia-Pacific, N. America	Both	55	29	26	100	0	0
Lab 19	Europe	LC-MS	40	40	–	0	0	100
Lab 20	Asia-Pacific, N. America	Both	23	12	11	4	9	87

### Methods and workflows used for LC-MS and GC-MS analysis

#### Extraction

The participants were instructed to use dichloromethane for extraction of dust for GC-MS analysis and dichloromethane:methanol (1:9, *v*/*v*) for extraction of dust for LC-MS analysis. The extraction technique and cleanup techniques were not specified, but all laboratories were requested to process a procedural blank in parallel to the sample.

#### LC-HRMS

An overview of LC-HRMS(MS) methods is presented in ESM Table S2 and Table S3. Most participants used C18 reversed phase columns with medium or very long UHPLC gradients. One of the 20 LC participants used a biphenyl column and one used a serial coupling of zwitterionic hydrophilic interaction (HILIC) and reversed phase (RP) chromatography (LC-LC). The solvent was typically water/methanol or water/acetonitrile (isopropanol as a second organic solvent in one case), either neat or with typical modifiers (e.g., formic acid or ammonium acetate/formate/fluoride). Between 2 and 35 μl of the extract was injected.

ESI was mainly used with different collision-induced dissociation (CID) or higher-energy CID (HCD) energies. Some participants submitted MS data only. One participant used atmospheric pressure chemical ionization (APCI) and atmosphere pressure photoionization (APPI) in addition to ESI. All participants who measured in both positive and negative ESI modes did so in separate runs. Some participants used “all-ion” data-independent acquisition approaches (fragmentation without precursor ion selection).

Most participants used time-of-flight (TOF), quadrupole TOF (QTOF), or ion mobility QTOF mass analyzers. Three used Orbitrap mass analyzers. The resolution of the TOF-based systems ranged from 10,000 to 42,000 and the resolution of the Orbitrap systems from 70,000 to 120,000.

In general, the most commonly used workflows consisted of peak-picking and deconvolution by instrument vendor software and spectra matching to commercially available or in-house mass spectral libraries. One participant used open-source XCMS [23] and R-packages, while another used the STOFF-IDENT open source platform [24]. MetFrag [25] was a popular tool used by many participants for prediction of MS/MS fragmentation. Two participants used correlation RT vs. log*D* to facilitate molecule identification.

#### GC-MS

An overview of GC-MS methods is presented in ESM Table S4 and Table S5. Most participants used nonpolar capillary columns coated with 5% phenyl polydimethylsiloxane or 5% phenyl dimethylarylene siloxane. One was using a 100% polymethylsiloxane column, and the remaining were using selectivity-tuned nonpolar columns (PAH, volatiles, EPA 1614). Two participants applied GC×GC, both using 50% phenyl columns for the second-dimension separation. All participants used hydrogen or helium as the carrier gas and all used total sample transfer techniques (splitless, PTV, or on-column injection). Roughly half the participants used speed optimized programs (6–10 °C/min) and the other half used efficiency-optimized oven temperature programs (2–5 °C/min). The former were generally employing target analysis workflows and the latter suspect and nontarget screening workflows.

A range of MS analyzers were used. The most popular were (Q)TOF analyzers, used by half of the participants, followed by triple-quadrupole (QQQ) and single-quadrupole analyzers, used by four and three participants, respectively. One data contributor used a GC-Orbitrap system. Most of the GC-(Q)TOFs were HRMS systems, ranging widely in age and performance. The reported mass resolution ranged from 5000 to 60,000 and the mass accuracy from 5 to 20 ppm, with one exception at 200 ppm. The remaining instruments were low-resolution mass spectrometers (LRMS) that provide unit mass resolution. Electron ionization (EI) was by far the most common ionization technique, used by all but one participant. Complementary data were generated in chemical ionization (CI) and APCI mode by four and two participants, respectively.

The most common suspect and nontarget screening workflow was peak-picking, EI library search (NIST and in-house libraries), manual spectra review, and (sometimes) interpretation. Several participants used peak and spectra deconvolution algorithms (PARAFAC, AMDIS, and similar) to enhance spectra quality. Some of these labs also used RI matching (NIST or in-house databases) to increase confidence in their identification. In the absence of RI data, at least one participant utilized RT correlation (RT vs. molecular weight) to check the plausibility of proposed structures. Additional information on the workflows and libraries used by each laboratory is given in Tables S3 and S5 of the ESM.

### Data curation

In nontarget screening, there is a clear risk for misassignment, especially for compound classes that produce very similar spectra and for compounds that do not produce strong molecular ion signals. The latter may, for instance, occur in LC-MS for compounds that easily produce stable adducts (and no molecular ions) and in GC-MS for compounds that easily fragment. Thus, proper data curation is essential to reduce the number of misassigned compounds that are added to the final list of compounds found in the house dust sample and to the indoor dust contaminant suspect list.

The level of data curation varied between the participants in the collaborative trial. Many participants applied in-house rules (basic to elaborate) for qualifying sample constituents for reporting and provided good documentation on what had been done. In several reported results, there were indications that some compounds had been misassigned, e.g., the same compound reported several times with different retention times, GC analyses with low molecular weight compounds reported with high retention indices, etc. It was therefore deemed necessary to perform additional curation of the reported data.

Expert evaluation of submitted LC-HRMS spectra and experimental or predicted RTI information were used to curate the contaminants found through suspect and nontarget screening analyses, thereby increasing the identification confidence.

## Results and discussion

### Curation of LC-MS data

Curation of the LC-MS data consisted of several steps. Only 50% volunteered to submit their raw chromatograms for further expert evaluation. Whenever MS/MS data were available for compounds reported with identification confidence level 3 or lower [26], and the participant did not include library searching in their workflow, a library search (MassBank, NIST, and Agilent commercial PCDL libraries) was performed. If no experimental spectra were available, MetFrag [25, 27] or CFM-ID [28] was used to predict possible MS/MS fragments of the reported compound. If the compound spectrum was found to agree with the library or in silico spectrum (minimum three fragments matching), it was added to the list of dust contaminants and was assigned identification confidence level 3. For example, one participant reported sorbitol monostearate, tri-xylenyl phosphate, ethyltriacetoxysilane, 2,2-dihydroxy-4,4-dimethoxy-benzophenone, acrylic acid, acrylic acid 2-ethylhexyl ester copolymer, phtalic acid divinyl ester, gallic acid propyl ester, *tert*-butyl-4-hydroxyanisole, and diacetoxy-di-*tert*-butoxysilane at level 4 confidence. However, the reported MS/MS spectra did not match the library or in silico spectra, and thus, those structures were likely incorrectly assigned and therefore were not included in the list of dust contaminants.

In the next step, data from participants that did not report MS/MS data were evaluated. Those results stem from suspect screening using exact mass (pseudo-molecular ion) information, which is error prone (i.e., high false-positive rate), unless carefully evaluated. To support such curation, calculated RTIs were employed. Two sets of calibration standards were used for indexing the LC-MS data. The first set was used as calibrants for QSRR models for RTI prediction, as described by Aalizadeh et al. [29]. The second set was used to establish a RTI/log*D* correlation within the FOR-IDENT platform (hosted at the Technical University of Munich, Germany) for compounds in the STOFF-IDENT database (Bavarian Environment Agency and the University of Applied Sciences Weihenstephan-Triesdorf, Germany). These resources may be found at https://www.lfu.bayern.de/stoffident and https://water.for-ident.org [24, 30]. In the FOR-IDENT processing workflow, the RTs of the candidate compounds are used to estimate their normalized retention time and thus correlated log*D* values (at a specific pH value). These observed log*D* values are then compared with the log*D* values for those molecules stored in the compound database STOFF-IDENT (Δlog*D*). In addition, in cases where alternative compounds with the same empirical formula are present in the STOFF-IDENT database, their Δlog*D* will also be calculated and all compounds will be ranked (original candidate and alternative compounds) in FOR-IDENT. Table S6 (see ESM) illustrates the application of RTI in the identification of some emerging contaminants reported by 12 participants, while ESM Table S7 shows an example of using Δlog*D* in FOR-IDENT platform for data curation.

A useful application of RTI is to remove false positives from the list of identified compounds. A compound is considered as false positive if it gives high residuals (error between predicted and experimental RTIs), while its structure belongs inside the application domain of the models, and the experimental RTI does not overlap with other participants or measurements. For participants that submitted calibration data for the FOR-IDENT platform and did not provide supporting evidence (i.e., no MS/MS, compounds not reported by other participants), all the compounds with a Δlog*D* outside ± 0.7 were considered as potential false positives. Compounds that fall within the acceptance window were assigned identification confidence level 3.

We cannot exclude the possibility that the LC columns used by participants are considerably different from those used to create the prediction models, resulting in a larger Δlog*D*. Fortunately, most laboratories in the current collaborative trial used LC columns similar to those used to create the model. However, some classes of compounds will undoubtedly lie outside the application domain of the predictive models. In such cases, the predictions are considered unreliable, but not necessarily wrong. This is illustrated by some of the surfactants, pentaethylene glycol, hexaethylene glycol, heptaethylene glycol, and octaethylene glycol that were reported by four participants. Although the structures were outside of the application domain of the RTI models, the RTIs on different LC systems were statistically equivalent and two participants reported the surfactants at identification confidence level 2a (mass spectra available in the library). Thus, those compounds were likely present in the dust. Less strict acceptance criteria should therefore be applied to this class of compounds.

#### Curation of GC-MS data

Alkanes were used for indexing of GC data. All participants used linear temperature programming, and therefore, the original Kovats equation (1958) was not applicable and the modified method by Van den Dool and Kratz [31] was used for calculation of linear retention indices (LRIs). These calculations were done by most of the participants. In the cases where no LRIs were reported, but retention time data for the alkanes was available, LRIs could still be calculated.

In a first round of data curation, the LRIs were correlated to the molecular weights of the reported dust contaminants. Compounds that were grossly deviating from the 1:1 line were removed. More than 100 compounds had to be removed and it was suspected that even more compounds were misassigned, but the regression model was too rough (*r*^2^ = 0.7 after removal of obvious outliers) to allow anything but a first rough curation. Attempts were made to improve the retention correlation model. A two-parameter regression model for LRIs vs. boiling points showed a stronger correlation, but there was still a considerable spread in data (*r*^2^ = 0.8). An Abraham general solubility model was therefore developed using Abraham constants from ACD labs Percenta software, with Absolve add-on (Toronto, Canada), and multiple linear regression (MLR) in Microsoft Excel. This model produced satisfactory results (*r*^2^ > 0.9) to allow recognition of suspected outliers. It proved difficult to set strict elimination criteria, mainly because some compound classes were poorly represented in the dataset and therefore likely outside the model domain. Potential outliers were therefore manually reviewed. A conservative approach was used, and compounds were only removed if there were strong reasons to do so, e.g., there was a large difference between the experimental and predicted LRIs and the compound was likely to be within the model domain.

**Fig. 1:**
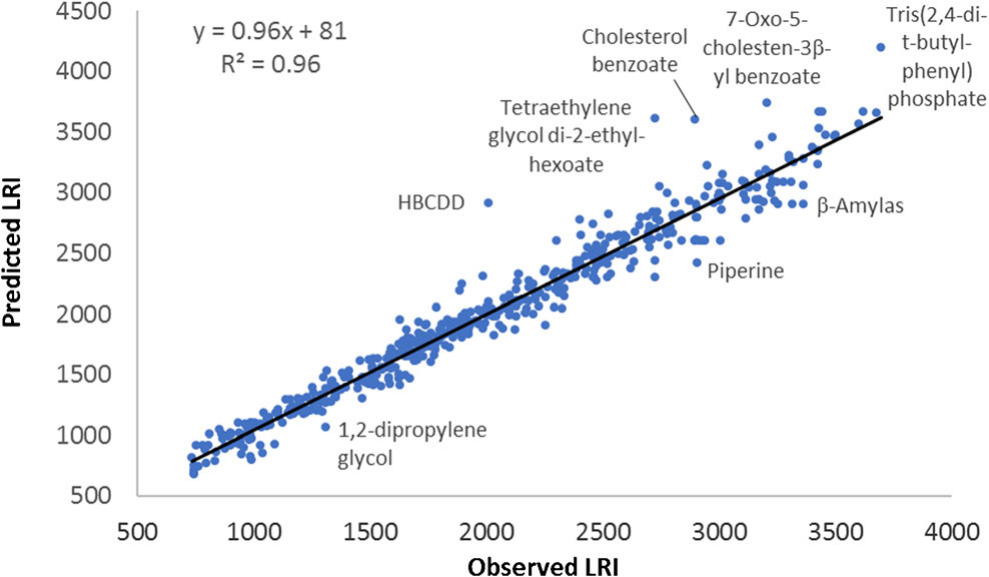
Graph of predicted and observed linear retention indices (LRIs) for the compounds remaining after curation of compound lists reported by GC-MS participants

A new Abraham model was constructed after elimination of compounds that were suspected to be misassigned. It exhibited a strong linear relationship with a slope close to 1 and an *r*^2^ of 0.96 (see Fig. 1). As can be seen, there were still compounds with LRIs deviating from the predicted LRIs, but those were generally target analytes or compounds that were reported by multiple laboratories (piperine, two glycols, HBCDD, and one organophosphate ester). There were some systematic deviations, e.g., cholesteryl benzoates were generally overestimated, and polyethylene glycols underestimated. In addition, all deviating compounds are likely to appear in indoor environments because they are constituents of food, personal care products, or building materials. The spread in the remaining LRI data is regarded as normal considering that the data was generated by multiple laboratories using a range of different nonpolar GC columns.

### Compounds identified in house dust

#### Database of identified compounds

**Fig. 2:**
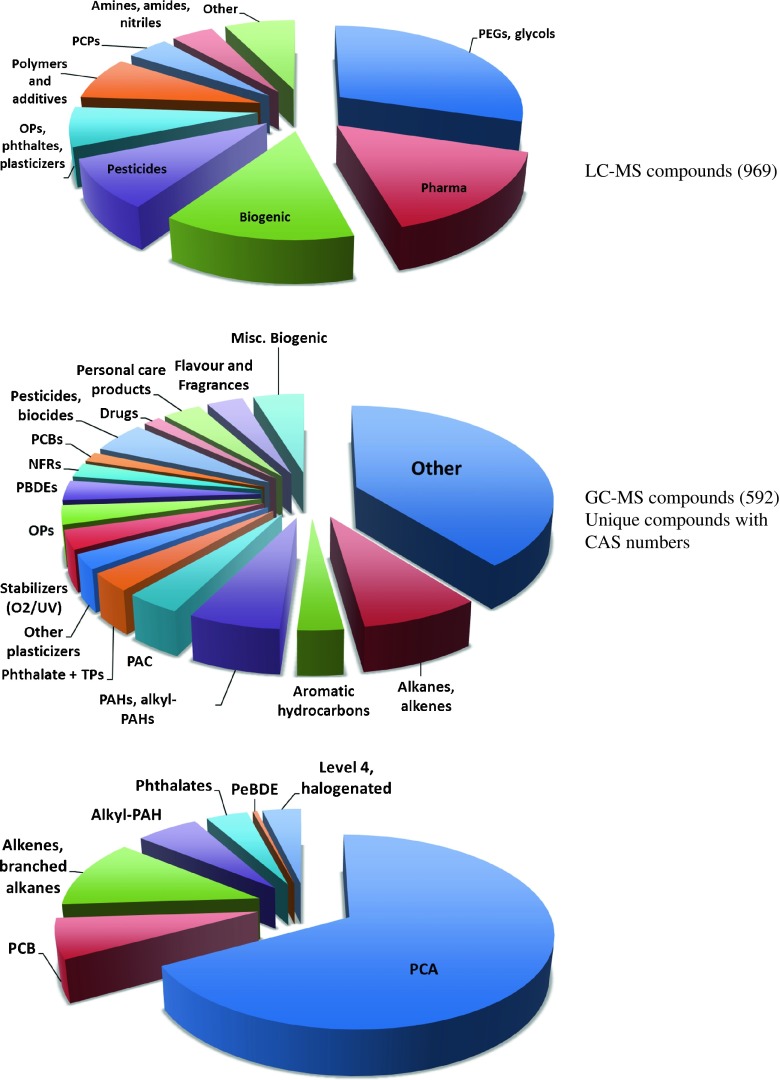
Overview of contaminant classes found in house dust using liquid chromatography (LC) and gas chromatography (GC)–mass spectrometry (MS) analysis

A compilation of the individual compounds that were identified or tentatively identified in house dust by the participants in the collaborative trial and that passed the data curation is provided in Microsoft Excel format as part of the ESM. The identification confidence level of each compound is given in the Excel file. Overall, nearly 2350 compounds were identified (18%) or tentatively identified (25% at confidence level 2 and 58% at confidence level 3). The compounds have been manually grouped based on origin (biogenic or anthropogenic), use category, or chemical class to aid a more detailed contaminant discussion. Figure 2 summarizes the major groups of contaminants found in house dust.

#### Compounds identified using LC-MS techniques

All participants using LC-MS techniques reported in total 969 compounds within the level of confidence 1–3 using the scale proposed earlier [26]. The contribution of individual participants was very variable between a few tens to several hundreds of compounds. Overall, only 59% of the reported compounds were identified or tentatively identified by more than one participant. Such differences most likely depend on the level of experience, time used for data evaluation, and probably factors such as access to libraries and suspect lists.

A wide range of different classes of compounds has been reported (Fig. 2) with glycols, phthalates, organophosphorus flame retardants, pharmaceuticals, biocides, and oxybenzone (UV-screen) being reported most frequently (5–10 times). Organophosphorus flame-retardant triphenyl phosphate (TPHP) and tris(2-butoxyethyl) phosphate (TBOEP) were the most frequently reported compounds by the LC-MS participants. TPHP is commonly found in target analyses of dust samples from different countries [32–37], and TBOEP is used in many indoor applications, for example, in plastics and floor polish [38].

Several biocides were reported and included imidacloprid, carbendazim, thiabendazole, and *N,N*-diethyl-m-toluamide (DEET). Imidacloprid is an insecticide belonging to the family of neonicotinoids. Carbendazim is a fungicide often used in house paints and plasterboards that continuously releases it into the environment. Together with imidacloprid, it was the most prevalent biocide in dust samples from Italy [39]. Thiabendazole is a fungicide used to control fruit and vegetable diseases such as mold, rot, blight, and stain. DEET is the most commonly used insect repellent and was previously reported as an indoor [1, 19] and outdoor air contaminant [40]. DEET is also detected in landfill leachate and drinking water [41].

Over 300 glycols and polyethylene glycol homologs (PEGs) have been detected with PEG-5 to PEG-16 being reported 5–10 times. PEGs are annually produced in millions of tons worldwide due to their broad use in cosmetics, plastic, water-soluble lubricants, pharmaceuticals, antifreeze agents, and nonionic surfactants [42].

#### Compounds identified using GC-MS techniques

The participants using GC-MS techniques reported in total 1281 compounds within the level of confidence 1–3 (Fig. 2). Most of the compounds (82%) were only reported by one participant. Many of these belong to the group of compounds that could not be assigned exact structure (because there were other positional isomers). Within the group of compounds that could be assigned exact structure and a CAS number, almost half (40%) were reported by more than one participant. For the remainder, the molecular formula and the backbone of the molecule could be established, but the exact location of the substituents was unknown, because the compounds had several positional isomers. For example, more than 450 medium chain–chlorinated paraffins were reported by one participant. The carbon chain length and degree of chlorination were known for all of them, but the carbon chain branching and chlorine substituent positions were unknown. Other compound classes with multiple positional isomers were PCBs and PBDEs (halogen position unknown), alkenes (double bond position unknown), branched alkanes and phthalates (branching unknown), and alkyl-substituted polycyclic aromatic hydrocarbons (alkyl-PAHs).

The reported compounds may be categorized into four major use/source categories accounting for 82% of all reported compounds: (1) persistent organic pollutants (POPs), (2) traffic-related compounds, (3) building material-related compounds, and (4) compounds related to pharmaceutical and personal care products (PPCPs). Persistent organic pollutants (medium chain polychlorinated paraffins (MCCPs), PCBs, PBDEs, and pesticides) accounted for 54% and traffic-related compounds (petroleum hydrocarbons, PAHs, and other PACs) for 25% of these compounds. The remaining compounds were distributed between building material and PPCP-related compounds, accounting for 11 and 9%, respectively.

Approximately 250 compounds did not fit into any of the major use categories and were therefore categorized according to chemical class. The major classes included: esters and ketones (23%), acids (17%), alcohols (15%), amides and amines (15%), aldehydes (15%), and miscellaneous compounds (14%).

The most frequently reported (i.e., 5–10 times) compounds belonged to plastics additives (22 compounds), PAHs (13 compounds), fatty acids (8 compounds), PPCPs (galaxolide, cetyl alcohol, and squalene), pesticides (permethrin and lambda-cyhalothrin), illicit drugs (cannabinol and delta-9-THC), and others (caffeine, cholesterol, vitamin E, and n-nonane).

The plastic additives included seven plasticizers (five phthalates, 2-ethylhexyl benzoate, and di-2-ethylhexyl adipate), seven organophosphate esters (TCEP, TCPP, TDCPP, TBP, TBEOP, TPHP, and EHDPP), six UV absorbers (benzophenone, oxybenzone, octyl salicylate, homosalate, octocrylene, and 2-ethylhexyl trans-4-methoxycinnamate), four polybrominated diphenyl ethers (BDE-47, BDE-99, BDE-100, BDE-153), and two antioxidants (BHT and 2,4-bis(1,1-dimethylethyl)phenol). Admittedly, some of the UV absorbers are also used in personal care products and could also have fallen into that category.

#### Compounds identified using both GC-MS and LC-MS techniques

There were few compounds reported by more than one participant, as mentioned before, and there was even a smaller share of the total number of compounds that were detected and reported by both LC-MS and GC-MS. Of the nearly 2400 tentatively identified compounds overall, only 5% were found by both techniques. The substances common to both platforms were predominantly fatty acids, glycols and polyethylene glycols, phthalates, and organophosphate esters. The remaining (minor) fraction measured by both platforms was as follows (in order of importance): amine/amides, pesticides, pharmaceuticals and illicit drugs, (nonphthalate) plasticizers, UV screens, PACs, and fragrances. Taken together, these data demonstrate that in order to comprehensively and holistically characterize contaminants in a complex matrix, such as house dust, it is necessary to use multiple LC-MS and GC-MS–based complementary analytical techniques.

### Use of complementary chromatographic and mass spectrometric techniques and workflows for nontargeted analysis of house dust

While several studies have identified numerous nontarget compounds with LC-ESI-HRMS, this approach alone does not provide a comprehensive picture of chemical contamination. Specific classes of environmental contaminants cannot be analyzed by this method due to inefficient ionization or incomplete separation. Therefore, alternative and complementary separation and ionization methods need to be applied to expand the range of nontarget screening. GC-MS is a necessary complementary technique for nonpolar compounds. Two-dimensional gas chromatography–mass spectrometry (GC×GC-MS) has proven to be an efficient tool for effective nontarget screening (NTS) of nonpolar compounds in the environment [14, 15]. It was also successfully applied by participants in the collaborative trial. Further, application of GC-HRMS with soft ionization methods such as methane CI or APCI can provide valuable molecular ion information and enables the use of LC-HRMS type of identification workflows, thereby providing complementary information to the established GC-EI-MS workflows.

**Fig. 3:**
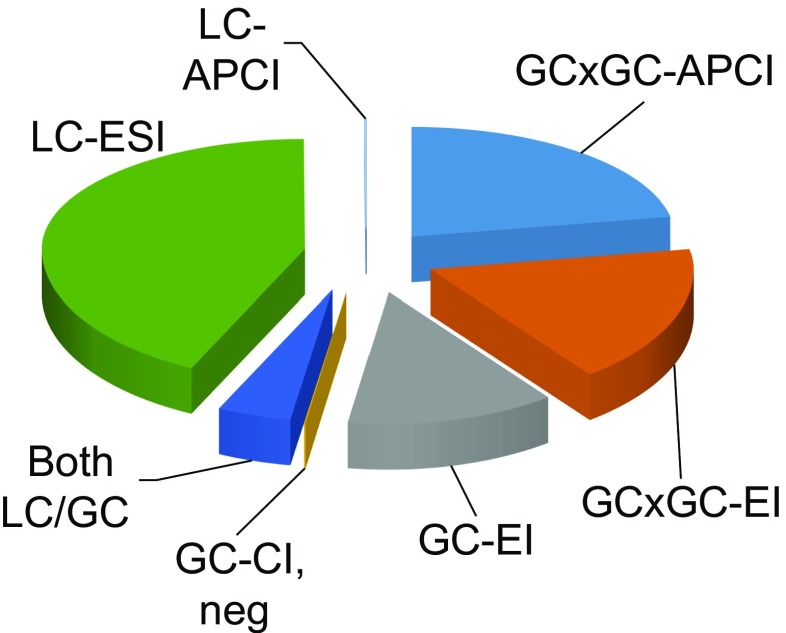
Distribution of the identified or tentatively identified compounds in house dust between the major instrument platforms used by the collaborative trial participants. Compounds that were detected by more than one technique were attributed to the platform contributing the largest number of compounds. Abbreviations: APCI, atmospheric pressure chemical ionization–mass spectrometry (MS); CI, neg, methane chemical ionization (CI) in negative ion mode; EI, electron ionization-MS; ESI, electrospray ionization-MS; GC, gas chromatography; LC, liquid chromatography

A breakdown of the (tentatively) identified compounds by the instrument platforms that have been applied by the participants in the collaborative trial is given in Fig. 3. It is clear that the use of four of the platforms (LC-ESI-MS, GC×GC-APCI-MS, GC×GC-EI-MS, and GC-EI-MS) has made a major contribution to the number of compounds identified in the house dust sample. A detailed discussion on the use of complementary separation and ionization techniques in LC-MS and GC-MS follows.

### Complementary ionization techniques and workflows in LC-MS

A comprehensive chemical identification can be maximized by using multiple ionization sources, such as ESI, APCI, and APPI in positive and negative modes. ESI mainly ionizes relatively polar compounds, while APCI can be employed to ionize less polar molecules. APPI is relatively less popular and used for nonpolar compounds, which are poorly ionized by ESI and APCI. ESI is the most widely used ionization technique, and by combining ESI in positive mode and ESI in negative mode, most compounds in an extract can be ionized. Complementary use of APCI and APPI enables the analysis of additional compounds, not ionized by ESI.

All three ionization techniques were used by participants in the collaborative trial. ESI in positive and negative ion modes was the most popular technique and was used to tentatively identify the greatest number of compounds. One of the participants applied APCI and APPI (positive and negative modes) and found 13 and 9 compounds, respectively. However, there was considerable overlap between the compounds observed with the different ionization techniques, and eight compounds were detected using all three sources. APCI in positive and negative modes identified two unique compounds, while APPI did not reveal any unique compounds.

### Complementary ionization techniques and workflows in GC-MS

#### GC×GC electron ionization MS

The GC-MS participant (Lab 2, Table 1) that reported most compounds (> 500) at an identification confidence of 2 or 3 used GC×GC-EI-MS. One of the main reasons for the high number of reported compounds was likely the high resolving power of GC×GC, which reduces the degree of background interference (i.e., co-elution). This results in cleaner MS spectra and better library match values, which ultimately improves tentative identification. Furthermore, the positions of the chromatographic peaks on the GC×GC plot are correlated to the physicochemical properties of the corresponding chemicals. In this case, the participant used a nonpolar (5% phenyl) column for the first-dimension separation and a semipolar (50% phenyl) column for the second-dimension separation. On such a column set, the first dimension of separation depends on volatility (as previously discussed), while the second dimension of separation depends on polarity. Thus, polar and polarizable compounds are retained more on the second-dimension column than nonpolar compounds. This information can be used as an additional discriminating factor when deciding whether to accept or reject a tentative structure. Collectively, this can allow detection and tentative identification of compounds present at relatively low concentrations, which are often associated with relatively low library match factors.

The ordered structure of GC×GC 2D contour plots also facilitates the identification of structurally related chemicals. The technique is, for example, increasingly used in the petroleum industry to perform PIONA: paraffins (alkanes), iso-paraffins (branched alkanes), olefins (alkenes), naphthenes (cyclic alkanes), and aromatics analysis. A group type separation is obtained in the second dimension, with paraffins eluting first, followed by olefins, naphthenes, monocyclic aromatics, bicyclic aromatics, and tricyclic aromatics [43]. In the first dimension, a separation based on carbon number is obtained. The iso-paraffins elute prior to the corresponding paraffins, in diagonal strikes, often referred to as the roof-tile effect [43].

Through the use of GC×GC, numerous petrogenic and pyrogenic hydrocarbons were tentatively identified and reported, including 50 alkanes, cycloalkanes, and alkenes; 18 monocyclic aromatic hydrocarbons; 43 PAHs; and 24 other polycyclic aromatic compound (PACs). In addition, 125 branched alkanes and alkyl-PAHs were reported. For the branched alkanes, the formula is known, but the degree and position of branching is unknown. Similarly, for the alkyl-PAHs, the formula and number of aromatic rings are known, but the type and position of substituents are unknown. These have therefore been reported as, e.g., C2-anthracene/phenanthrene.

**Fig. 4:**
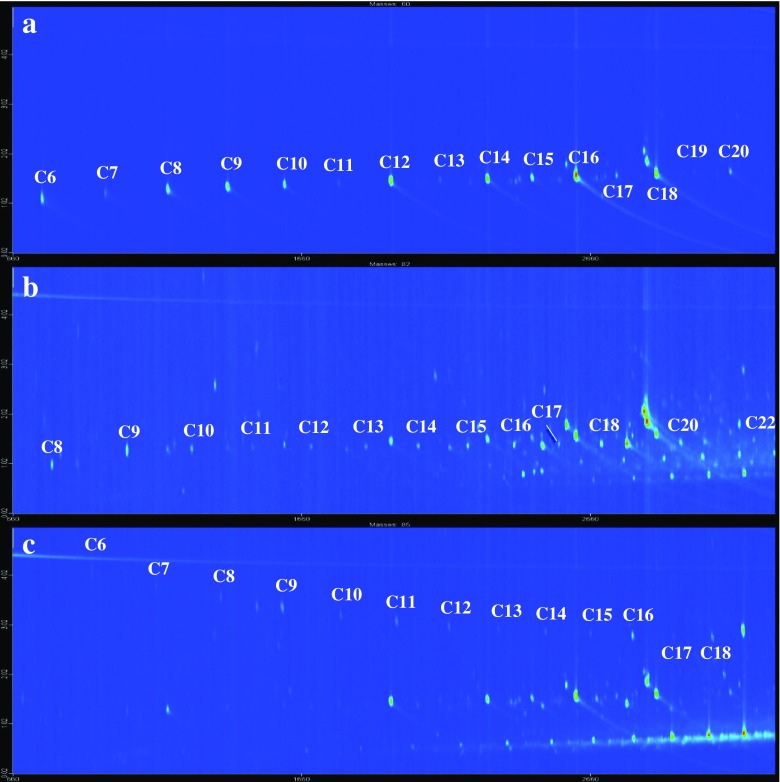
Extracted ion chromatograms (*m*/*z* values in parentheses) from comprehensive 2D gas chromatography analyses of house dust, illustrating the ordered elution patterns of three homologous series of dust contaminants: **a** aliphatic (*n*-alkyl) acids (*m*/*z* 60), **b** aliphatic (*n*-alkyl) aldehydes (*m*/*z* 82), and **c** cyclic aliphatic lactones (*n*-alkyl furanones) (*m*/*z* 85)

The structured 2D chromatograms make it relatively straightforward to tentatively identify sample constituents belonging to other homologous series of compounds, even if not all of these are represented in commercial EI-MS libraries. Figure 4 shows extracted ion chromatograms (EICs) of diagnostic ions of aliphatic acids, aldehydes, and lactones. Through the detection and characterization of a few members of each class of compounds all members of the respective homologous series can be tentatively identified. In addition to the compounds included in Fig. 3, homologous series of alkylamides, *N,N*-dimethylamines, n-alkanols, and PEGs were tentatively identified and reported by GC×GC laboratories.

#### GC×GC atmospheric pressure chemical ionization HRMS

**Fig. 5:**
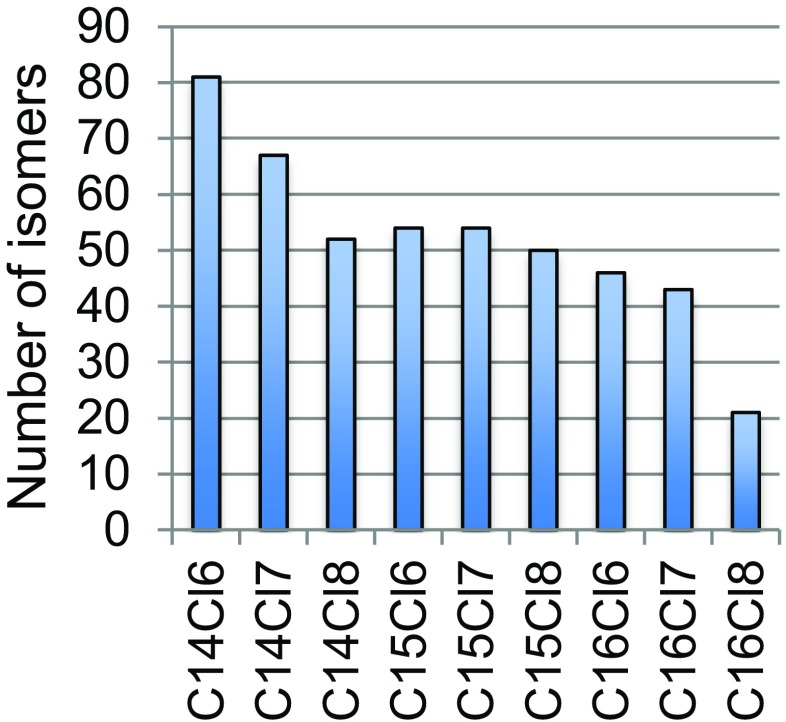
Homolog pattern of medium chain–chlorinated paraffins detected in house dust using comprehensive 2D gas chromatography and negative ion atmospheric pressure chemical ionization mass spectrometry (GC×GC-APCI(−)-MS)

One of the participants (Lab 3, Table 1) used GC×GC-APCI-HR-TOF-MS in both positive and negative ion modes and was able to detect and tentatively identify more than 500 halogenated chemicals. Sixty-five compounds were found using APCI in positive ion mode, including 9 pesticides, 13 organophosphorus and brominated flame retardants, 33 PCBs, and 8 halogenated compounds for which only the formula could be generated. The remaining compounds (468) were all found using APCI in negative ion mode and were all MCCPs. The MCCPs were detected as oxygen adducts, i.e., as [M+O_2_]^−^. The homolog pattern is shown in Fig. 5. It is somewhat surprising that no short chain polychlorinated paraffins (SCCPs) were detected as those have previously been reported in house dust [44].

Chlorinated paraffin (CP) mixtures are exceptionally complex and the use of GC×GC greatly facilitates the separation of CP homolog groups as well as isomers, as demonstrated by Korytár et al. [45]. Using this technique, the separation is much improved over 1D GC, and an ordered structure with diagonally arranged peaks is obtained, containing CPs differing in the number of chlorines. There are, however, still overlaps among CPs with the same number of halogens and different numbers of carbons. When combined with a soft ionization technique, such as APCI, a complete characterization of chlorinated paraffin mixtures can be achieved.

GC-APCI-MS has recently attracted attention and has proven valuable in NTS studies [46]. Its main advantage over GC-EI-MS is that molecular ions or molecular ion adducts are usually obtained, which enables the possibility of using an LC-MS/MS type of NTS workflow for the identification of unknowns. In addition, it may provide an attractive option for laboratories that have access to LC-HRMS instrumentation, but no dedicated GC-HRMS instrumentation.

#### GC-LRMS with complementary full-scan EI and methane PCI and NCI

The main advantage of using a GC coupled with a single quadrupole mass spectrometer (in addition to the relatively low cost and availability) is that there are several easily exchangeable ionization techniques available that provide complementary information and increase the number of compounds that can be tentatively identified with a high level of confidence. One participant used three types of ionization EI, and methane-positive ion chemical ionization (PCI) and positive ion chemical ionization (NCI), all in full-scan mode. The identification workflow was based on the full-scan EI data and the use of mass spectral and LRI databases (allowing a ± 50 RI unit tolerance). Often this was sufficient for a tentative identification; however, many compounds displayed chimeric (mixed) spectra or spectra without a clear molecular ion. In such cases, the PCI and NCI data provided useful complementary information, PCI provided molecular ion or adduct ion information for compounds with high proton affinity, and NCI provided molecular ion information for compounds with high electron affinity and high molecular ion stability. NCI can also be used to verify the presence of electronegative atoms, mainly halogens, through inspection of chlorine and bromine EICs. These compounds can sometimes be tentatively identified by matching with entries in a “home-made” NCI library. Three examples of the complementary use of EI, CI, and LRI information are given in the following sections (i) verification of candidate structure, (ii) correction of proposed structure, and (iii) tentative identification based solely on NCI data.

In the first example, a NIST library search indicated the presence of two isomers of cyhalothrin (MW = 449) in the dust extract. The total ion chromatogram (TIC), full-scan spectrum, and base peak chromatogram (*m*/*z* 181) are shown in ESM Fig. S1. The base peak chromatogram displays two peaks, indicated with yellow arrows, potentially corresponding to stereoisomers. However, no molecular ions could be found for verification, even after manual extraction. Instead, the pseudo-molecular mass (*m*/*z* 450) of cyhalothrin isomers was confirmed through the use of the PCI result (window B) and NCI confirmed the presence of chlorine. The good agreement of the experimental LRI (2576) and NIST RI (2579) further strengthened the proposed structure.

The second example is illustrated by ESM Fig. S2. The top panel displays an EIC (*m*/*z* 163) with three peaks labeled with yellow arrows. Their manually extracted spectra were very similar and one of them is shown (panel B). A NIST search offered *N*-propyl benzamide as the most likely compound (86% match), but the calculated LRI (2485) did not match that of NIST (1526) indicating a misassignment. After applying some constraints (e.g., size), another candidate was found, dipropyleneglycol dibenzoate (three isomers), which had a much better LRI match (2445). Because the dipropyleneglycol dibenzoate EI spectrum lacks a molecular ion, PCI was used for verification and a pseudo-molecular ion (MH) was found. Dipropyleneglycol dibenzoates had also been detected by other participants, which further strengthens the tentative identification.

**Fig. 6:**
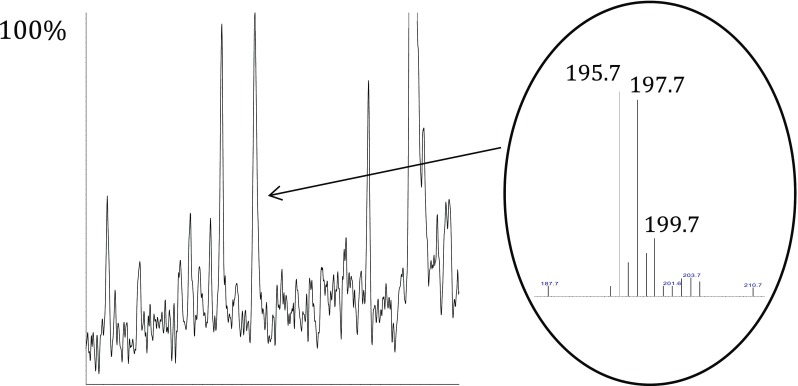
Negative ion chemical ionization total ion chromatogram (left) and the manually extracted spectrum of one of the peaks (right), tentatively identified as a trichlorophenol

The final example is illustrated by Fig. 6. An unknown compound was observed at retention time 15.29 min in the TIC from the NCI analysis of the dust extract. Its full-scan spectrum showed the typical characteristic chlorine isotope distribution pattern of compounds containing three chlorine atoms. After searching a “home-made” NCI library, 1,3,5-trichlorophenol was found to be the closest match. It should have a LRI of 1335 according to NIST and the experimental RI was 1371, which supports the proposed peak assignment, although other trichlorophenol isomers cannot be ruled out. Inspection of the EI and PCI chromatograms did not reveal any signals in the relevant LRI range that could be attributed to trichlorophenols. This is most likely because the response of those is lower in EI and PCI than in NCI, which further corroborates the value of using complementary ionization techniques.

### Factors influencing detection potential in suspect and nontarget screening of house dust

#### Physical–chemical properties of compounds detected by GC- and LC-MS

High complementarity between the techniques used by the participants in the collaborative trial was observed (Fig. 3). GC-MS participants reported mainly small non- and semipolar molecules (ESM Fig. S3, left side), while semipolar and polar compounds dominated the LC-MS results (ESM Fig. S3, right side). The compounds that were reported following both GC-MS and LC-MS analyses have, in general, intermediate size and polarity, as expected.

All LC-MS participants were using positive ion ESI. A few were also using negative ion ESI, APCI, or APPI. However, application of APPI only resulted in the discovery of two unique compounds. This does not mean that a majority of LC-MS-amenable compounds in dust respond well in positive ion ESI. On the contrary, several environmental contaminants respond well in negative ion ESI, APCI, or APPI, but poorly in positive ion ESI [47]. Thus, there is likely a bias in the results toward compounds with high proton affinity, which respond well in positive ion ESI. This may explain the high percentage of compounds with high basicity, such as amines, amides, nitriles, and polyethers (incl. glycols), among the LC-MS datasets.

Among the GC-MS techniques, the use of 2D separations and complementary soft (CI) and traditional (EI) ionization proved valuable. Two participants using GC×GC-APCI-MS and GC×GC-EI-MS, respectively, collectively found more than 1000 compounds. The use of CI did not, in isolation, lead to identification of many compounds, mainly due to lack of commercial CI spectral libraries. However, GC-PCI-MS still proved valuable as it provided molecular ion information that could confirm candidate structures obtained using GC-EI-MS. Similarly, negative ion CI was used to confirm the presence of halogen substituents. This technique has also been used to guide discovery workflows aimed at finding and identifying halogenated environmental contaminants [48]. The PCI and NCI soft ionization techniques are in themselves complementary, as they selectively ionize compounds with high proton affinity and high electron affinity, respectively.

In principle, the techniques used by the participants cover a large part of the chemical domain of contaminants likely to be found in house dust. However, very polar compounds are not sufficiently covered. These require a separate LC-MS analysis employing, e.g., a HILIC column, which was only used by one participant. Consequently, only a few compounds with log *K*_ow_ values below zero were reported (ESM Fig. S3). Similarly, large nonpolar compounds with molecular weight above 600 Da were also insufficiently covered. The largest hydrocarbon n-alkane detected was n-hexatriacontane (506.6 Da), the largest nonhalogenated compound was tri(2-ethylhexyl) trimellitate (546.4 Da), and the largest of all nonpolar compounds was decabromodiphenyl ether (961.2 Da). However, analysis of the latter was done by targeted methods aimed at brominated flame retardants, utilizing short thin-film GC columns.

#### Lack of conformity among reported datasets

Almost half of the compounds reported from LC-MS analyses and most of the compounds reported from GC-MS analysis were only reported once.

The lack of agreement in GC-MS results may be attributed to differences in applied methodology and instrumentation. A large share of the results was generated using GC×GC-EI-MS and GC×GC-APCI-MS which differs in selectivity. In addition, many compounds separated by GC×GC would co-elute in 1D-GC, making identification much more difficult. An important share of the unique compounds was also detected using targeted approaches, which usually are more sensitive and selective than nontargeted approaches.

The LC-MS results are more difficult to explain. Almost all compounds were identified using the same technique, LC-ESI(+)-HRMS/MS. A question then arises—Is the small overlap between data generated using similar hardware due to differences in data handling? Most likely, this is part of the reason. However, it is also likely that the experience and time invested by the participants have significantly influenced the outcome.

Almost all LC-MS data originated from target analysis or suspect screening approaches. If we look at the self-reported categorization of workflows, 14% of the reported data was generated using target analysis, 46% using a suspect screening workflow, and 40% using a NTS workflow. However, only one third of the compounds that were self-classified as NTS data had supporting MS/MS information, which is generally required for a tentative identification. Of the remaining 187 compounds, 11 were related to organophosphate and phthalate esters, which had been listed as suspects by the organizers, and 125 were related to glycols that are well-known contaminants in house dust. Besides these, 50 compounds (3% of all reported) were generated using NTS workflows that included MS/MS confirmation.

Consequently, the majority of the confirmed and tentatively identified compounds stem from suspect screening using candidate lists or searches of ESI mass spectral libraries. Different groups have access to different suspect lists and different mass spectral libraries, which often are vendor specific. Obviously, this influenced the compounds identified by the various participants. This may also be one of the major reasons for the poor overlap between the LC-MS and GC-MS datasets. The LC-ESI-MS libraries are usually relatively small and rich in compounds relevant for the life sciences, pharmaceutical research, and environmental and forensic toxicology, while the GC-EI-MS libraries are large, more diverse, and also include a large range of industrial chemicals. Thus, many of the reference mass spectra in the LC-MS libraries are missing in GC-MS libraries, and vice versa.

## Guidelines to successful screening and reporting of contaminants in indoor environment samples

### Using feasible suspect lists

One of the main outcomes of the collaborative effort is the generation of an extensive database with house dust contaminants. More than 2300 compounds were identified or tentatively identified of which close to 1700 could be assigned exact chemical structures. The database greatly expands our knowledge base of contaminants in house dust. As a comparison, a recent compilation of house dust contaminants by Zhang et al. [49] included a total of 485 compounds, including ca 250 compounds from an earlier compilation by Mercier et al. [50]. In addition, a GC×GC-MS screening of contaminants in house dust revealed 10,000 peaks of which 370 could be characterized (145 PAHs, 52 phthalates, 8 nitro compounds, and 165 chlorine/bromine-containing compounds) [20]. A more recent study using both LC-QTOF-MS and GC-QTOF-MS reported 271 house dust contaminants of which 163 could be unambiguously confirmed by reference standards [21].

The list of dust contaminants generated in the collaborative trial will be amended with additional compounds from the above cited studies and with indoor air contaminants reported by members of the NORMAN Association. This will result in a NORMAN indoor environment contaminants suspect list, which will be shared through the NORMAN suspect list exchange program. NORMAN partners are also working on compiling and curating a master list termed “SusDat” containing information needed in NTS workflows for screening of known environmentally relevant compounds (both lists can be found at www.norman-network.net, “databases” tab). Next to unique identifiers such as StdInChIKey, MS Ready InChIKey, SMILE, and QSAR model-predicted ecotoxicological limit values, exact masses of expected adduct ions in both positive and negative ionization modes and related RTIs are now available for more than 40,000 substances (as of October 2018). Work is currently in progress on obtaining experimental and predicted mass fragments for all compounds to support identifications of these suspects in the NORMAN Digital Sample Freezing Platform (DSFP; www.norman-data.eu).

The “CompTox Chemistry Dashboard” at the U.S. Environmental Protection Agency (U.S. EPA) is an even larger well-curated repository of compound information. It contains many potential candidate lists, including the NORMAN lists, but also contains useful additional data to support identification, such as physicochemical properties, literature references, patent data, functional uses, and (eco)toxicological data. NORMAN and U.S. EPA closely cooperate on further development of SusDat and the two databases are interlinked.

The above and other similar initiatives are in progress to expand the support of suspect screening and NTS workflows, including MS spectra predictions, and prioritization of compounds found in such studies. A recent paper by Rager et al. [22] illustrates the potential of such platforms for combined LC-MS suspect screening analysis, exposure and toxicity prediction, and ranking of dust contaminants. An even more recent paper by Phillips et al. [51] describes the use of GC×GC-MS for suspect screening analysis of chemicals in consumer products, which are highly relevant as sources for contaminants in house dust.

### Using complementary chromatographic and ionization techniques

Access to dedicated suspect lists through NORMAN, U.S. EPA, and other web resources will make it possible to better utilize the information generated by less commonly used ionization techniques, such as CI, APCI, and APPI, and thereby increase the coverage of contaminants in indoor environment samples. It will also increase the overlap of the chemical domains covered by LC-MS and GC-MS techniques. Detection and tentative identification of compounds by two or more independent analytical method greatly enhances the identification confidence.

The parallel use of EI and PCI may yield library searchable spectra and molecular ion information, which, when combined with RI information, can result in a level 2 identification confidence. The use of NCI and negative ion APCI and ESI can be used to selectively screen for halogenated compounds [52], which often cause environmental concern. Furthermore, APCI is generally less sensitive to matrix effects than ESI and, therefore, useful for semiquantification of LC-MS-amenable compounds.

Based on the results of the collaborative trial, the most powerful combination of instrumental techniques seems to be ESI-HRMS/MS and GC×GC-EI-(HR)MS. Most of the reported compounds stem from either of those techniques. However, with the easy access to tailored suspect lists, it may prove fruitful in the future to complement those techniques with one or more CI techniques. Many top-end LC-HRMS systems allow both LC-APCI and GC-APCI analysis to be performed on the same platform, which may be worth exploring further.

Although not tested in the collaborative trial, liquid chromatography–based multidimensional techniques (e.g., 2D liquid chromatography (LC×LC) MS and LC ion mobility MS/MS) and supercritical fluid chromatography (SFC) are also expected to provide enhanced separation power and peak capacity. Current developments in multidimensional data evaluation software may unravel the full potential of such techniques.

### Using retention indices to enhance confidence in identification

Suspect screening using suspect lists and molecular formula generated using LC-HRMS information will, initially, have a high rate of false positives. The number of false positives can be subsequently reduced using various discriminators. One of the most effective discriminators is chromatographic retention indices. An automated routine was developed by partners of the NORMAN network to predict LC RTIs using a set of calibration compounds [29] or use it experimentally for normalization and prioritization of candidate molecules by correlated log*D* values [24, 30], and this was subsequently used in the present work for LC-MS data curation. The approach utilizes a set of carefully chosen molecular descriptors and quantitative structure–retention relationships to predict the RTIs of candidate structures based on the retention times of the calibrants while at the same time determining if the individual candidates are within the model domain. Work is currently in progress to generate RTIs for all compounds on the SusDat list which, once fully implemented, will greatly facilitate the suspect qualification process.

Although the NIST library of EI-MS spectra includes GC RI information, it is not always easy to use. Some participants in the collaborative trial did not appear to use this information, which resulted in misassignments. Some MS software allows the use of RI information for ranking of the NIST spectral search hit list, which reduces the probability of false positives. However, even if the NIST database contains more than 72,000 compounds, RIs are still lacking for many compounds encountered in a complex matrix, such as house dust. A simple QSRR, such as the Abraham general solubility model developed for curation of the collaborative trial, may then be used for prediction of LRIs. In many cases, a sufficiently good prediction can be achieved by a single-parameter retention model, using linear regression of the GC retention times vs. the analyte vapor–hexadecane partition coefficients (Abraham *L* coefficient). A linear regression model created using the curated GC-MS data had an *r*^2^ = 0.95.

For GC×GC data, two independent retention times (or indices) are available that can be used to discriminate between potential candidates and to reduce the percentage of false positives. Methods for GC×GC retention indexing and prediction have recently been developed and tested [53, 54].

### Using in silico–predicted mass spectra and metadata to enhance the identification confidence

Several participants used in silico tools (MetFrag, MSC (Molecular Structure Correlator), and Mass Frontier) for fragment confirmation, thereby raising the identification confidence. In this context, the development of automatic routines for suspect list and database curation and generation of “MS-ready” structures is of importance [55]. These tools have been adopted in the CompTox database and the substances have been desalted, desolvated, and had stereochemistry removed to represent the forms of chemicals observed via HRMS. Easy access to curated MS-ready structures will greatly facilitate suspect screening of known unknowns. In the future, the NORMAN SusDat and the Dashboard records may be linked to those from open spectral libraries (e.g., MassBank and MoNa) and fragmentation prediction resources (e.g., MetFrag, CFM-ID, and Mass Frontier) to further streamline the process to raise the identification confidence [56].

In addition, further ranking of candidate chemicals using data source ranking or functional use filtering has proven effective. The latest CASMI (Critical Assessment of Small Molecule Identification) challenge showed that the success rate of high-throughput (semiautomated) identification routines could be increased from 34 to 70% by including metadata (www.casmi-contest.org/2016/). Data source ranking (number of PubChem references or patents) is supported in MetFrag [25] and data source statistics (total number of data sources and of PubChem records/data sources), and product occurrence data (EPA CPCat) is available through CompTox Dashboard [57]. Chemical use and function category data, organized with descriptors such as detergent, food-additive, etc., are also available in the dashboard. These data may support tentative chemical identification through filtering by the use category relative to the sample medium—here dust. Further development to create a weighting-based or tiered ranking approach for identification using the aforementioned criteria as inputs is underway [57].

### Using open-source data processing platforms

The poor overlap between the GC-MS and LC-MS-derived datasets from individual participants is hypothesized to be mainly influenced by the varying experiences of the laboratories, the time spent for analyzing the data, and varying access to tools such as mass spectral libraries, dust-relevant suspect lists, and data-processing tools, rather than instrumental limitations. This hypothesis was confirmed after uploading selected LC-MS data to the recently developed Norman DSFP and perform suspect screening using the complete list of compounds identified in the study. Briefly, the dust LC-MS raw data files (from different vendors) were converted to mzML format, imported to DSFP together with instrumental metadata, contributor details, and retention times of the retention index calibrants. An internally standardized procedure of peak-picking and componentization (using previously optimized parameters) is utilized to create a component list, which then can be matched against any list of suspected substances considering their exact mass, fragmentation, and retention time plausibility through QSRR RTI models. The tentative results were obtained in a short time and the number of identified compounds exceeded on average 500. Additional compound lists can be found at the NORMAN Suspect List Exchange (https://www.norman-network.com/?q=node/236). Suspect screening against all the compounds in the NORMAN SusDat database allowed for provisional identification of additional 476 compounds not reported by the participants.

The FOR-IDENT platform offers another open access tool (https://water.for-ident.org), as previously discussed. It uses retention time/RTI, accurate mass/empirical formula, and mass spectra information of uploaded (suspects or nontarget screening) mzML files or raw data files from LC-MS vendors, which are compared with the information of compounds in the STOFF-IDENT database (formula, log*D*, and in silico fragmentation spectra from the MetFrag tool and MassBank entries). The FOR-IDENT platform gives on this basis molecular identification suggestions with prioritization levels.

### Using harmonized terms and identification levels when reporting untargeted analysis data

From the results of the collaborative trial, it is clear that not even established users of untargeted analytical techniques have a common use of the terms “target,” “suspect,” and “nontarget” analysis and the identification confidence levels 2–5 [16, 26]. It seemed to be a more coherent view on the terms and levels among the LC-HRMS community, which is logical. That community was instrumental in the development of a common nomenclature and identification confidence system. In future reporting of untargeted analysis data, LC-HRMS users are encouraged to strictly follow the guidelines laid out by Schymanski et al. [16].

The identification confidence scheme [16, 26] appeared less easily applicable for the GC-MS participants, most likely due to differences in preferred workflows. Level 5 (molecular weight known) and level 4 (formula known) make little sense to most GC-MS users. They work directly with library search results and must decide when to accept the top hit from library, when to pick another candidate, and when to reject all candidates (and potentially peruse a true nontargeted workflow). Guidance is needed on what is required to qualify a provisional structure for level 3 (plausible structure) and level 2 (probable structure), respectively. Work is currently underway to formulate such guidelines.

## Electronic supplementary material

ESM 1 (PDF 2.86 mb)

ESM 2 (XLSX 231 kb)
