# Supplementary material for: The strength in numbers: comprehensive characterization of house dust using complementary mass spectrometric techniques
Source: Anal Bioanal Chem. 2019 Mar 4;411(10):1957–77. doi: 10.1007/s00216-019-01615-6 (PMC6458998; doi:10.1007/s00216-019-01615-6)
Supplement: Supplementary file 1 — (PDF 2.86 mb) [file 216_2019_1615_MOESM1_ESM.pdf]

Analytical and Bioanalytical Chemistry

Electronic Supplementary Material

The strength in numbers: comprehensive characterization of house dust using complementary mass spectrometric techniques

Pawel Rostkowski, Peter Haglund, Reza Aalizadeh, Nikiforos Alygizakis, Nikolaos Thomaidis, Joaquin Beltran Arandes, Pernilla Bohlin Nizzetto, Petra Booij Hélène Budzinski, Pamela Brunswick, Adrian Covaci, Christine Gallampois, Sylvia Grosse, Ralph Hindle, Ildiko Ipolyi, Karl Jobst, Sarit L.Kaserzon, Pim Leonards, Francois Lestremau, Thomas Letzel, Jörgen Magnér, Hidenori Matsukami, Christoph Moschet, Peter Oswald, Merle Plassmann, Jaroslav Slobodnik, Chun Yang

Additional file available under 10.1007/s00216-019-01615-6

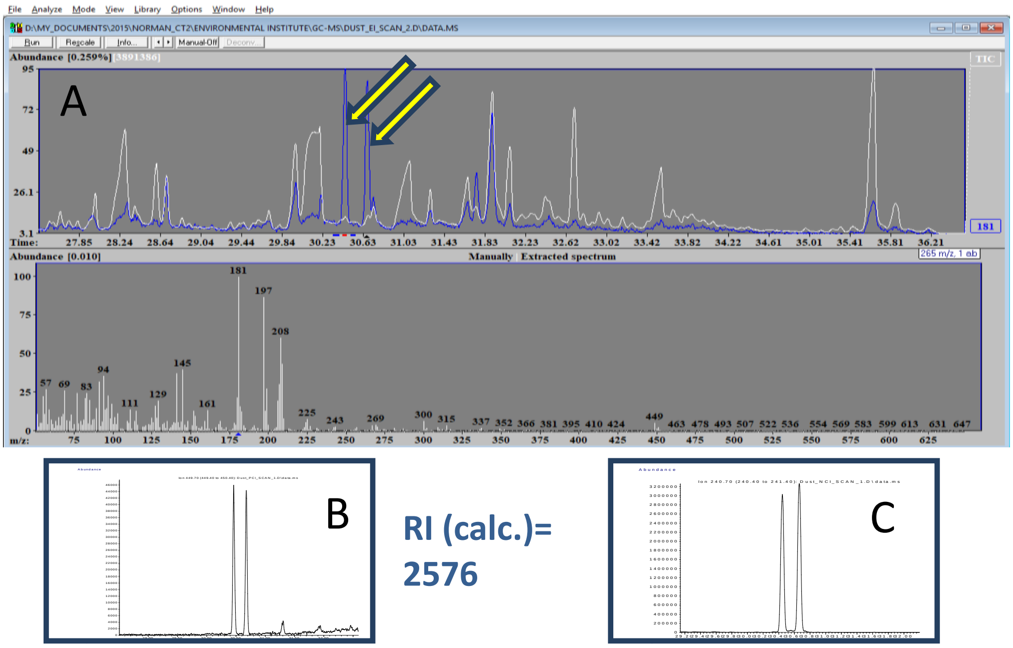

Fig. S1 Tentative identification of cyhalothrin in house dust extract. A) Total ion chromatogram (TIC), electron ionization (EI) spectrum, and base peak chromatogram (m/z 181). The base peak chromatogram displays two peaks, indicated with yellow arrows that were tentatively identified as cyhalothrin isomers. B) Positive chemical ionization (PCI) mass fragmentogram of cyhalothrin pseudo-molecular ions (m/z 449.7). C) Negative chemical ionization (NCI) mass fragmentogram of a characteristic cyhalothrin fragment (m/z 240.7), demonstrating an excellent signal-to-noise ratio as a result of high response in NCI because of the presence of halogens (at m/z 240.7)

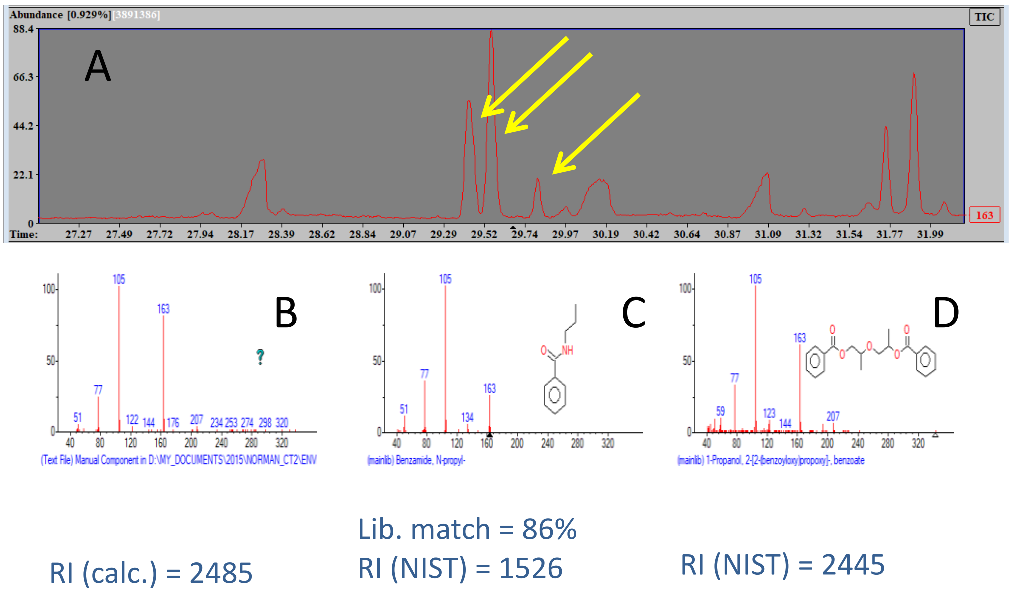

Fig. S2 Tentative identification of dipropyleneglycol dibenzoates in house dust extract. A) Extracted ion chromatogram (m/z 163) with three peaks labelled with yellow arrows. B) Manually extracted spectrum of one of the three peaks (all spectra very similar). C) NIST electron ionization (EI) spectrum of the best matching compound N-propyl benzamide (match 86%). D) Electron ionization (EI) spectrum of an alternative compound dipropyleneglycol dibenzoate; obtained after applying constraints. The retention index (RI) of sample constituent “B” and the two NIST entries are shown below each spectrum

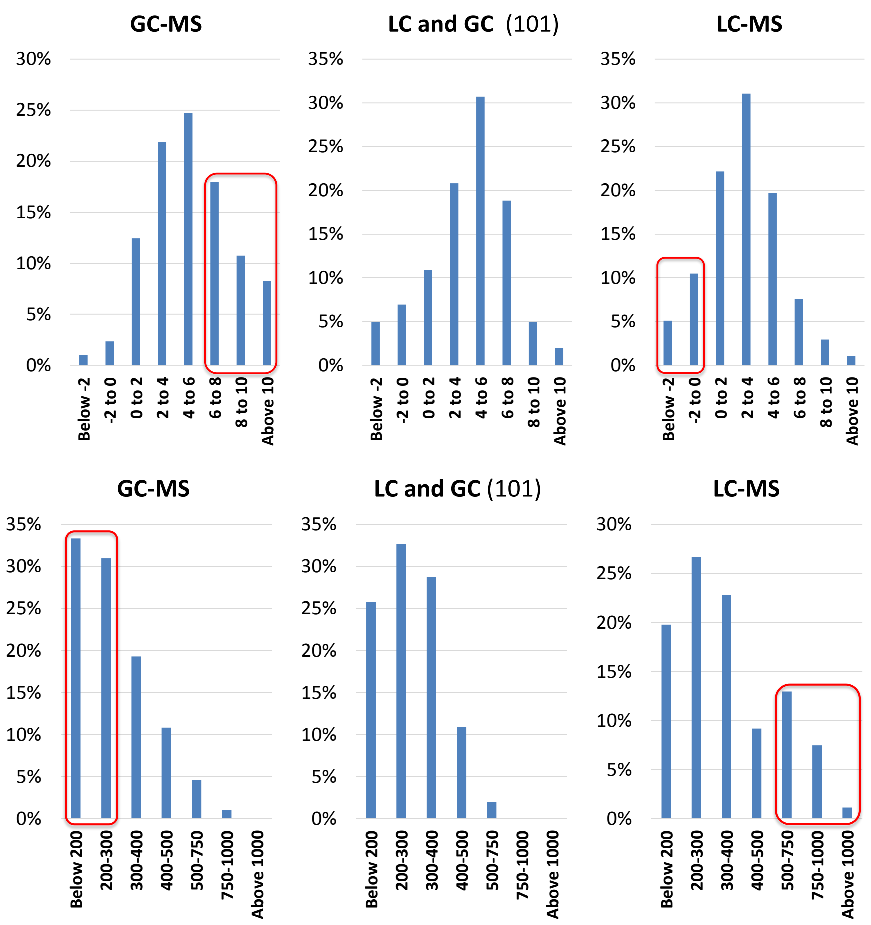

Fig. S3 Lipophilicity distribution (top row) and size distribution of (tentatively) identified compounds in house dust

Table S1a Information about the compounds used for the calculation of RTI for the LC-MS techniques. LogP was calculated using ChemAxon

**Substance name**	**LogP**
Metformin	-1.36
Chloridazon	1.11
Carbetamide	1.65
Monuron	1.93
Metobromuron	2.24
Chlorbromuron	2.85
Metconazole	3.59
Diazinon	4.19
Quinoxyfen	4.98
Fenofibrate	5.28

Table S1b Information about the compounds used for the calculation of RTI for the LCMS techniques (TrAMS RTI model)

Type of calibrant	Substance name	CAS number
ESI+	Guanylurea	141-83-3
ESI+	Amitrol	61-82-5
ESI+	Histamine	51-45-6
ESI+	Chlormequat	999-81-5
ESI+	Methamidophos	10265-92-6
ESI+	Vancomycin	1404-90-6
ESI+	Cefoperazone	62893-19-0
ESI+	Trichlorfon (Dylox)	52-68-6
ESI+	Butocarboxim	34681-10-2
ESI+	Dichlorvos	62-73-7
ESI+	Tylosin	1401-69-0
ESI+	TCMTB	21564-17-0
ESI+	Rifaximin	80621-81-4
ESI+	Spinosad A (Spinosyn A)	131929-60-7
ESI+	Emamectin B1a	121124-29-6
ESI+	Avermectin B1a (Abamectin)	71751-41-2
ESI+	Nigericin	28380-24-7
ESI+	Ivermectin B1a	70288-86-7
ESI-	Amitrole	61-82-5
ESI-	Benzoic acid	65-85-0
ESI-	Acephate	30560-19-1
ESI-	Salicylic acid	69-72-7
ESI-	Simazine 2-Hydroxy	2599-11-3
ESI-	Tepraloxydim	149979-41-9
ESI-	Bromoxynil	1689-84-5
ESI-	MCPA	94-74-6
ESI-	Valproic acid	99-66-1
ESI-	Phenytoin	57-41-0
ESI-	Flamprop	58667-63-3
ESI-	Benodanil	15310-01-7
ESI-	Dinoterb	1420-07-1
ESI-	Inabenfide	82211-24-3
ESI-	Coumaphos	56-72-4
ESI-	Triclosan	3380-34-5
ESI-	AvermectinB1a (Abamectin)	65195-55-3
ESI-	Salinomycin	53003-10-4

Table S2 Information about the LC-MS equipment and LC conditions used by the participants in the collaborative trial

Participant number	Instrument and model	Column	Dimensions	Solvent	Injection (µL)	Flow (ml/min); run time (min)
Lab 1	Agilent 6550 Q-TOF	Agilent Poroshell 120 SB-C18	3.5 µm 2.1x50mm	positive mode: A= 0.1% FA, B= ACN, 0.1% FA negative mode: A= 0.1% AcA, B= ACN, 0.1% AcA	2	0.4; 55
Lab 2	Agilent 6560 IM-QTOF	Phenomenex	2.6 µm 2.1x150mm	A= 0.1% FA B= MeOH, 0.1% FA	1	0.5; 60
Lab 4	Agilent 6530 Q-TOF	Zorbax Eclipse Plus C18	1.8 µm 2.1x100mm	positive mode: A= 0.1% FA, B=ACN, 0.1% FA Negative mode: A= 1mM NH4F B=ACN	10	0.35; 22
Lab 5	Agilent 6550 Q-TOF	Acquity HSS T3	1.8 µm 2.1x50mm	A= 10nM NH4Ac, pH 4.7 FA B= MeOH	50	0.4; 20
Lab 6	Bruker Maxis Impact	Acclaim RSLC C18	2.2 µm 2.1x100mm	A= 10% MeOH, 0.01% FA, 5mM ammonium formate B= MeOH, 0.01% FA, 5mM ammonium formate	5	0.2-0.48; 20
Lab 7	Agilent 6550 Q-TOF	Waters Acquity HSS T3	1.8 µm 2.1x150	A= water B= MeOH	3	0.3, 30
Lab 8	Exactive Orbitrap LC-MS	Agilent, Poroshell C18	2.7 µm 2.1x50mm	A= water, B= ACN C= Isopropanol	5	0.2; 40
Lab 9	Agilent 6550 Q-TOF	Phenomenex Kinetex-EVO C18	2.6 µm 2.1x100mm	A= 10mM NH4Ac B= MeOH	2	0.4; 25
Lab 10	Waters XEVO G2 QTOF	Waters Cortecs C18	3.5 µm 2.1x100mm	A= 0.01% FA B= MeOH, 0.01% FA	20	0.3; 14
Lab 11	Agilent 6545 QTOF	Agilent Poroshell 120 Phenyl-Hexyl	2.7 um 3.0x100mm	A = 1 mM NH4F B = MeOH	5	0.4; 16
Lab 12	Q Exactive HF Orbitrap	Hypersil GOLD aQ (Thermo) C18 (aq)	1.9 µm 2.1x100mm	A= 0.1% FA, B= ACN, 0.1% FA	5	0.4; 10
Lab 13	Agilent 6530 Q-TOF	Phenomenex Kinetex Biphenyl	2.6 µm 2.1x100mm	A= 0.04% FA, pH 2.6 B= 95 % ACN, 5 % A	2	0.4; 25.5
Lab 14	Bruker micro TOF II	GL Sciences InertSustain C18	3.5µm 2.1x100mm	A: water B: MeOH	0.3	
Lab 15	Q Exactive Focus Orbitrap	Thermo Accucore C18	2.6 µm 2.1x50mm	A= 10mM HOAc B= MeOH	2	0.3; 21
Lab 16	Agilent 6540 Q-TOF	Phenomenex Kinetex C18	1.7 µm 2.1x100mm	positive mode: A= 0.1% FA, B= ACN, 0.1% FA negative mode: A=water; B=ACN	2	0.3; 47
Lab 17	Agilent 6550 Q-TOF	Waters Acquity UPLC BEH C18	1.7µm 2.1x100mm	A= 10%MeOH, 0.1% FA B= MeOH, 0.1%FA	2	0.3; 22.5
Lab 18	Agilent 6530 Q-TOF	Zorbax Eclipse Plus C18	1.8 µm 2.1x100 mm	A= 5mM NH4Ac B = 5mM NH4Ac in MeOH	2	0.3; 45
Lab 19	Agilent 6230 TOF	C18 and Zic-HILIC	5 µm, 2.1x150mm (HILIC), 2.7 µm 3.0x50mm (C18)	A= CAN, B= water (HILIC), A= 90% 10mM NH4Ac: 10% ACN, B= 90% ACN: 10% 10mM NH4Ac (C18)	10	0.05 (C18); 0.4 (HILIC)
Lab 20	AB Sciex Triple TOF 5600	Agilent Zorbax XDB C18	ESi+ : 1.8 µm, 2.1x100mm ESI- : 1.8 µm, 4.6x50mm	positive mode: A= 0.1% FA, B= MeOH, 0.1% FA negative mode: A= 1% MeOH, 5nM NH4Ac B= 95% MeOH, 5mM NH4Ac	ESI+: 5 ESI-: 10	ESI+: 0.4, 16.50 ESI-: 10

Table S3 Information about the MS conditions and workflows used by the LC-MS participants in the collaborative trial

Participant number	Scan Range	Resolution	Ionization	Fragmentation method	Target Software	Suspect, NT procedure
Lab 1	110-1100	42,000 (m/z=922)	ESI	NA (MS only)	Mass Hunter	Mass Hunter, in-house library, Find by formula
Lab 2	50-1700	40,000 (m/z=922)	ESI	CID	Mass Hunter	Mass Hunter, Forensic Tox, Melin, in-house library
Lab 4	50-1050	10,000 (m/z=600)	ESI	CE	Agilent Mass Hunter Quant	Agilent Mass Hunter Qual, MS, MS/MS library, MS/MS prediction, correlation RT vs. log D (Jchem), R2=0.70, Agilent Molecular Structure Correlator
Lab 5	100-1000	Ca 40,000	ESI	CID	Mass Hunter Suite	PCDL Forensic & Tox and Pesticides, MetFrag, MassFrontier
Lab 6	50-1000	35,000 (m/z=500)	ESI	CID	TASQ 1.0	xcms+nontarget R-packages
Lab 7	100-1700	12,000 (m/z=119)	ESI	CID	Mass Hunter	Mass Hunter, Agilent Forensic Tox, NILU suspect database
Lab 8	120-1200	100,000 (m/z=200)	ESI		Xcalibur	ExactFinder
Lab 9	50-1700	25,000 (m/z=1000)	ESI	CID	Mass Hunter	Mass Profiler
Lab 10	50-1000	20,000 (m/z=556)	ESI	CID	ChromaLynx XS	MS, RT, MSE, prediction, MassFragment, MetFrag, Mass Frontier
Lab 11	20-1700	21,000 (m/z 622)	ESI	NA (MS only)	Mass Hunter	Metlin, In-house library
Lab 12	100-1000	120,000 (m/z=200)	ESI	HCD	Xcalibur	Compound Discoverer, mzCloud and MetFrag
Lab 13	59-1100	10,000 (m/z=200)	ESI	CID	Mass Hunter	Mass Hunter Qual, METLIN, MetFrag, manual fragmentation
Lab 14	100-1000	16,500	ESI		Target Analysis (Bruker)	RT prediction on logP, number of references in Chemspider
Lab 15	75-1000	70,000 (m/z=200)	ESI	AIF	Trace Finder	
Lab 16	50-1000	12,000 (m/z=113) 21,000 (m/z=602)	ESI	CID	Mass Hunter	Mass Hunter, Forensic Tox, Melin, MassBank, MetFrag, Pesticides
Lab 17	50-1000	Ca 40,000	ESI, APCI, APPI			
Lab 18	100-1500	Ca 10,000	ESI	NA	Mass Hunter Qual	
Lab 19		10,000 (m/z 1700)	ESI	NA	NA	Profinder, STOFF-IDENT
Lab 20	100-1000	30,000 (m/z=956)	ESI	CID	PeakView (MasterView)	isotopic pattern, distribution, double bonds and rings, different chemical DBs (chemspider etc)

Table S4 Information about the GC-MS equipment and GC conditions used by the participants in the collaborative trial

Lab	Instrument and model	Column[a]	Dimensions	Injection Volume	Temperature program	Carrier gas/flow (ml/min)	IS[b]
Lab 1	Agilent 7000A<br>GC-QQQ	DB-PAH	30 m, 0.25 mm, 0.15 µm	SSL<br>2 µL	50C (2min)-2.35C/min-310C (8 min)	He<br>1.0	D8-NAPH, D10-ACEN/PHEN, D12-PER/CRYS
Lab 2	EI: Leco Pegasus 4D<br>GC×GC-QTOF<br>CI:Agilent 7200B<br>GC-QTOF	Rtx-5sil ms<br>BPX-50<br>DB-5ms UI	30 m, 0.25 mm, 0.25 µm<br>2 m, 0.18 mm, 0.18 µm<br>30 m, 0.25 mm, 0.25 µm	SSL<br>1 µL	35C (1 min)-4C/min-310C (3 min)<br>60C (1 min)-4C/min-335C (3 min)<br>90(1)-4C/min-310C (3 min)	He<br>1.0<br>He<br>0.8	D10-PHEN
Lab 3	Waters Xevo G2-XS<br>GC×GC-QTOF	Rtx-5<br>Rtx-17	60 m, 0.25 mm, 0.25 µm<br>1 m, 0.15 mm, 0.15 µm	SSL<br>1 µL	90C (2 min)-40C/min-200C-2.8C/min-330C (5min)	He<br>2.0	^{13}C-PBDE mix
Lab 4	Agilent 7200B<br>QTOF	DB-5ms UI	30 m, 0.25 mm, 0.25 µm	SSL<br>1 µL	35C(3 min)-4C/min-325 (3 min)	He<br>1.0	DBOFB
Lab 5	Agilent 5795C<br>GC-MS	DB-5ms	30 m, 0.25 mm, 1.00 µm	LVI,<br>20 µL	50C (2min)-8C/min-320C (10 min)	H_2<br>1.8	D10-chlorpyrifos<br>D10-PHEN
Lab 7	Agilent 7200 Q-TOF<br>GC 7890B	HP-5MS UI	30 m, 0.25 mm, 0.25 µm	PTV<br>1µl	45(2 min)- 310°C	He<br>1.2	-
Lab 8	Agilent 7200<br>GC-QTOF	DB-5ms	30 m, 0.25 mm, 0.25 µm	SSL<br>1 µL	40C (1 min)-5C/min-310C (25 min)	He<br>1.0	D14-Terphenyl
Lab 9	Agilent 5793<br>GC-MS	EI: ZB-volatiles<br>NCI: Rxi-PAH	30 m, 0.25 mm, 0.50 µm<br>30 m, 0.25 mm, 0.1 µm	PTV<br>1 µL	EI: 40C (5 min)-5C/min-320C (5 min)<br>NCI: 70C (5 min)-45C/min-190C-5C/min-320 (5 min)	He<br>1.2	Deuterated mix: nitro-PAH and quinone-PAH
Lab 10	Xevo G2, GC-QTOF<br>Shimadzu 2010, GC-MS<br><br>Waters TSQ, GC-QQQ	DB-5ms<br>DB-5ms<br><br>DB-HT1	30 m, 0.25 mm, 0.25 µm<br>30 m, 0.25 mm, 0.25 µm<br>15 m, 0.25 mm, 0.10 µm	SSL<br>1 µL	90C (0 min)-5C/min-315C (4 min)<br>60C (3 min)-10C/min-240C-30C/min-320 (5 min)<br>140C (1 min)-10C/min-200C-20C/min-300C-40C/min-350C (1 min)	H_2, 2.0<br>H_2, 2.0<br><br>H_2, 4.0	D8-DDT
Lab 13	Agilent 7200<br>GC-QTOF	HP-5ms	30 m, 0.25 mm, 0.25 µm	SSL<br>1 µL	90C (2 min)-20C/min-200C-5C/min-290C (32 min)	He<br>1.0	–
Lab 16	Agilent 7200<br>GC-QTOF	Rtx-1614	30 m, 0.25 mm, 0.10 µm	SSL<br>1 µL	50C (2 min)-2C/min- 310C (10 min)	He<br>1.0	–
Lab 17	GC-Q Exactive<br>GC-Orbitrap	TG-5sil-ms	30 m, 0.25 mm, 0.25 µm	SSL<br>1 µL	80C (1 min)-7C/min-310C (2 min)	He<br>1.0	–
Lab 18	Agilent 7000C<br>GC-QQQ	DB-5ms	15 m, 0.25 mm, 0.10 µm	On-column<br>2 µL	100℃ (1 min)-10℃/min-310℃ (10 min)	He<br>1.0	^{13}C-PBDE mix
Lab 20	Thermo TSQ<br>GC-QQQ	DB-5ms	30 m, 0.25 mm, 0.25 µm	SSL<br>1 µL	80C (2 min)-20C/min-180C-10C/min-300C (10 min)	H_2<br>1.0	DCDD

[a] Column manufactures: DB: J&W, HP: Agilent, Rtx: Restek, BPX: SGE, TG: Thermo, ZB: Phenomenex,
Abbreviations: PHEN: Phenanthrene, ACEN: Acenaphthalene, Per: Perylene, CRYS: Crysene, DBOFB: 4,4'-dibromo-octaflurobiphenyl.

Table S5 Information about the MS conditions and workflows used by the GC-MS participants in the collaborative trial

Lab	Mass Range	Resolution, mass accuracy	Ionization	Target Software	Suspect, NT procedure	RI mixture
Lab 1	50-600	Unit	EI	Mass Hunter	Mass Hunter, manual interpretation	Yes
Lab 2	29-750<br>30-1000	Unit<br>12,000<br>5 ppm	EI<br>CI(–)	–<br>–	ChromaTOF, NIST14, manual interpretation, RI matching, RT correlation, Chemspider<br>Masshunter, Br^{-} and Cl^{-} detection, full-spectrum extraction, manual review, RI matching, Chemspider	Yes
Lab 3	50-1200	25,000<br>5 ppm	APCI(+)<br>APCI(–)	–	GCImage v2.5	No
Lab 4	50-1050	8,400<br>15 ppm	EI	Mass Hunter Quantitative	Masshunter Qualitative incl. Unknown Analysis, RI matching	Yes
Lab 5	45-800	Unit	EI, CI(+) and CI(–) (CH_4)	Chemstation, Amdis, NIST14	Library search, RI matching, manual interpretation	Yes
Lab 7	50-1000	2ppm, 16-17000 FWHM	EI	Mass Hunter Quantitative	Mass Hunter Unknown Analysis, Library Search (NIST 17, Agilent Pesticides), manual interpretation, RI matching	Yes
Lab 8	50-750	10,000<br>5 ppm	EI	Mass Hunter	Mass Hunter, manual interpretation	Yes
Lab 9	EI: 50-800<br>NCI: 50-400	Unit	EI,<br>CI(–)	Chemstation	Chemstation, Amdis, NIST11	Yes
Lab 10	QTOF: 50-650<br>GC-MSD: 50-650<br>GC-QQQ: MRM	12,000<br>Unit<br>Unit	APCI<br>EI<br>APCI	Chromalynx, Shimadzu GCMSsolution	Chromalynx, PARAFAC, NIST search, manual interpretation	Yes
Lab 13	50-1000	8,000<br>20 ppm	EI	–	Masshunter, NIST	Yes
Lab 16	50-1000	8,000<br>10 ppm	EI	–	Masshunter Qualitative incl. Unknown Analysis, NIST, manual interpretation	Yes
Lab 17	66-1000	60,000<br>10 ppm	EI	Tracefinder,<br>In-house library	–	No
Lab 18	MRM	Unit	EI	Mass Hunter Quant	–	Yes (C16-C30)
Lab 20	50-700	Unit	EI	–	NIST search	Yes

Table S6 Practical application of RTI for improving identification of emerging pollutants

No.	*multiple comparison procedure plot*	*RTI details*
(1)	see plot below	see table below

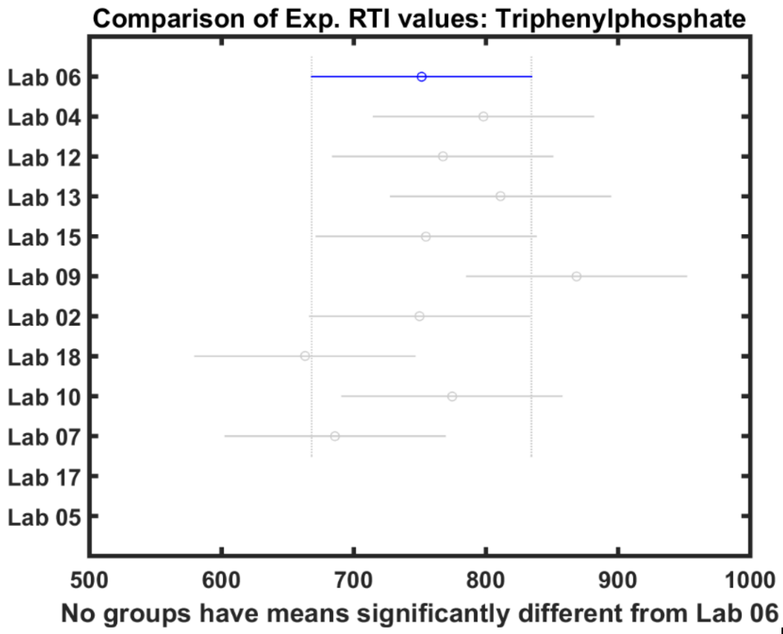

Institute	Level of identification confidence	Exp. t_R (min)	Exp. RTI	Pred. RTI	ESI mode	Application domain	MCP*
Lab 6	1	11.15	751.27	740.51	+ESI	AD1	a
Lab 5	N.D.	N.D.	N.D.	N.D.	N.D.	N.D.	N.D.
Lab 4	2	13.85	776.40	740.51	+ESI	AD1	a
Lab 12	2a	7.426	767.26	740.51	+ESI	AD1	a
Lab 13	3	21.58	811.13	740.51	+ESI	AD1	a
Lab 15	1	9.11	754.82	740.51	+ESI	AD1	a
Lab 9	2	14.96	848.45	740.51	+ESI	AD2	a
Lab 2	1	33.42	749.83	740.51	+ESI	AD1	a
Lab 18	1	14.91	663.04	740.51	+ESI	AD1	a
Lab 17	N.D.	N.D.	N.D.	N.D.	N.D.	N.D.	N.D.
Lab 10	2	12.52	774.28	740.51	+ESI	AD1	a
Lab 7	2	16.67	685.85	740.51	+ESI	AD1	a

AD1: Exp. & Pred. tR are accepted for this candidate (box1)
AD2: Although there is error, exp. & pred. tR are accepted for this candidate (box2)
N.D.: Not detected.
*MCP is multiple comparison procedure; the difference in exp. RTI values is encoded as letter (RTI values are significantly different if they have different letters (e.g. “a” vs “b”)
All institutes have acceptable exp. RTI values and the difference is **not significant at 99%CIs** of the calibration curve. **Promote “Lab 13” to level 2!**

No.	multiple comparison procedure plot	RTI details

(2)

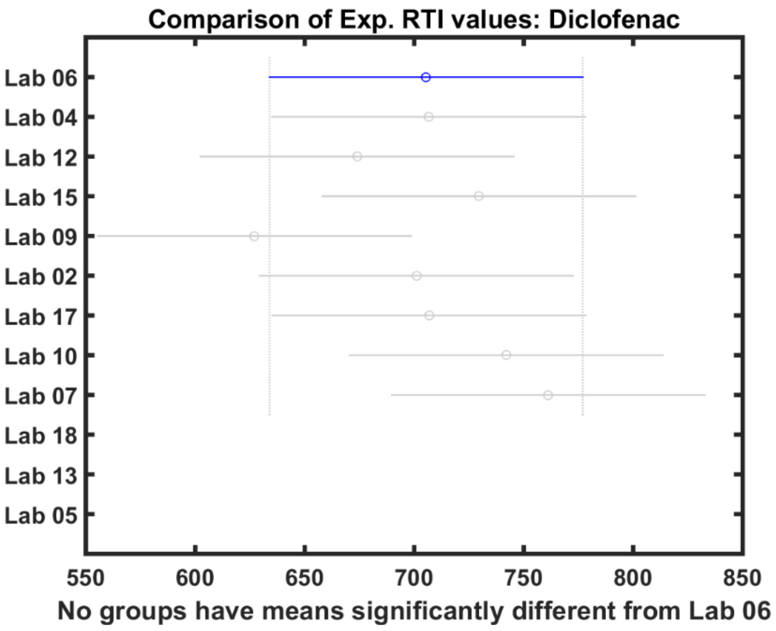

Institute	Level of identification confidence	Exp. t_R (min)	Exp. RTI	Pred. RTI	ESI mode	Application domain	MCP
Lab 6	1	10.54	705.44	725.40	+ESI	AD1	a
Lab 5	N.D.	N.D.	N.D.	N.D.	N.D.	N.D.	N.D.
Lab 4	2	12.42	687.24	725.40	+ESI	AD1	a
Lab 12	2a	6.58	673.92	725.40	+ESI	AD1	a
Lab 13	N.D.	N.D.	N.D.	N.D.	N.D.	N.D.	N.D.
Lab 15	1	8.81	729.63	725.40	+ESI	AD1	a
Lab 9	1	11.54	619.67	725.40	+ESI	AD2	a
Lab 2	2	31.01	700.98	725.40	+ESI	AD1	a
Lab 18	N.D.	N.D.	N.D.	N.D.	N.D.	N.D.	N.D.
Lab 17	1	12.55	706.78	725.40	+ESI	AD1	a
Lab 10	1	12.01	741.98	725.40	+ESI	AD1	a
Lab 7	2	18.11	761.20	725.40	+ESI	AD1	a

AD1: Exp. & Pred. tR are accepted for this candidate (box1)
AD2: Although there is error, exp. & pred. tR are accepted for this candidate (box2)
N.D.: Not detected.
All institutes have acceptable exp. RTI values and the difference is **not significant at 99%CIs** of the calibration curve.

(3)

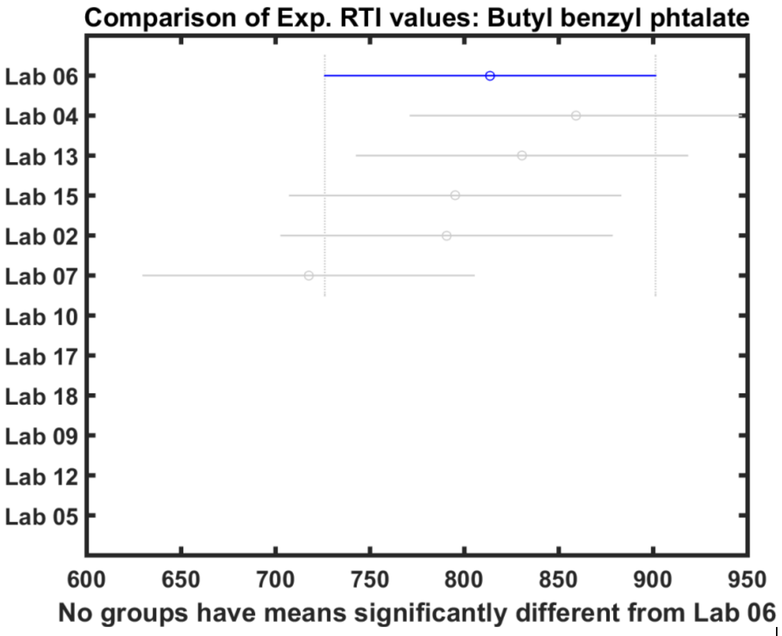

Institute	Level of identification confidence	Exp. t_R (min)	Exp. RTI	Pred. RTI	ESI mode	Application domain	MCP
Lab 6	1	11.96	813.64	728.09	+ESI	AD1	a
Lab 5	N.D.	N.D.	N.D.	N.D.	N.D.	N.D.	N.D.
Lab 4	2	14.80	835.59	728.09	+ESI	AD2	a
Lab 12	N.D.	N.D.	N.D.	N.D.	N.D.	N.D.	N.D.
Lab 13	4	22.08	830.54	728.09	+ESI	AD2	a
Lab 15	1	9.59	795.11	728.09	+ESI	AD1	a
Lab 9	N.D.	N.D.	N.D.	N.D.	N.D.	N.D.	N.D.
Lab 2	1	35.43	790.58	728.09	+ESI	AD1	a
Lab 18	N.D.	N.D.	N.D.	N.D.	N.D.	N.D.	N.D.
Lab 17	N.D.	N.D.	N.D.	N.D.	N.D.	N.D.	N.D.
Lab 10	N.D.	N.D.	N.D.	N.D.	N.D.	N.D.	N.D.
Lab 7	2	17.28	717.49	728.09	+ESI	AD1	a

AD1: Exp. & Pred. tR are accepted for this candidate (box1)
AD2: Although there is error, exp. & pred. tR are accepted for this candidate (box2)
N.D.: Not detected.
All institutes have acceptable exp. RTI values and the difference is **not significant at 99%CIs** of the calibration curve. **Promote “Lab 13” to level 2!**

No.	*multiple comparison procedure plot*	*RTI details*

(4)

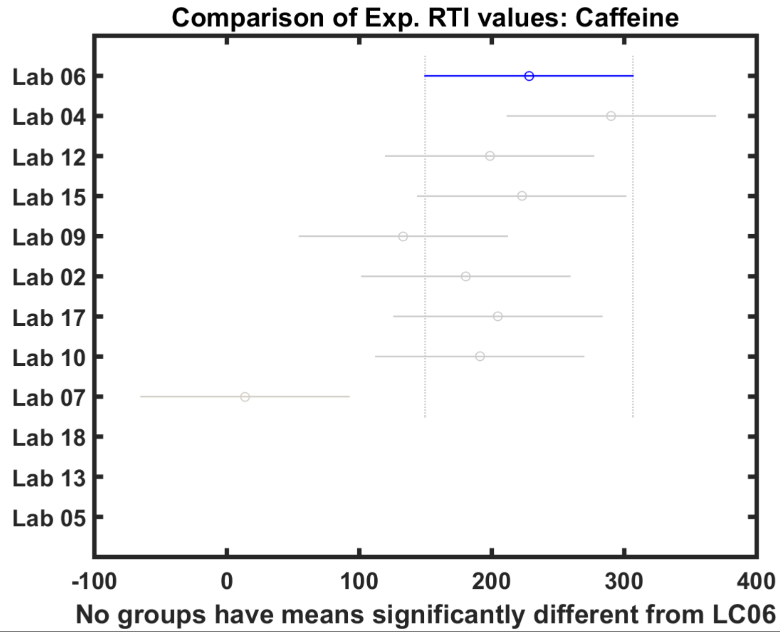

Institute	Level of identification confidence	Exp. t_R (min)	Exp. RTI	Pred. RTI	ESI mode	Application domain	MCP
Lab 6	1	4.29	228.10	224.24	+ESI	AD1	a
Lab 5	N.D.	N.D.	N.D.	N.D.	N.D.	N.D.	N.D.
Lab 4	2	5.92	282.24	224.24	+ESI	AD1	a
Lab 12	2a	2.25	198.40	224.24	+ESI	AD1	a
Lab 13	N.D.	N.D.	N.D.	N.D.	N.D.	N.D.	N.D.
Lab 15	1	2.77	222.63	224.24	+ESI	AD1	a
Lab 9	2	4.54	151.96	224.24	+ESI	AD1	a
Lab 2	2	5.33	180.39	224.24	+ESI	AD1	a
Lab 18	N.D.	N.D.	N.D.	N.D.	N.D.	N.D.	N.D.
Lab 17	1	4.01	204.65	224.24	+ESI	AD1	a
Lab 10	2	3.31	190.93	224.24	+ESI	AD1	a
Lab 7	1	2.15	13.76	224.24	+ESI	AD3	a

AD1: Exp. & Pred. tR are accepted for this candidate (box1)
AD3: Pred./Exp. tR are not reliable (high SR; use other verification tools (box3))
N.D.: Not detected.
All institutes have acceptable exp. RTI values and the difference is **not significant at 99%CIs** of the calibration curve.

(5)

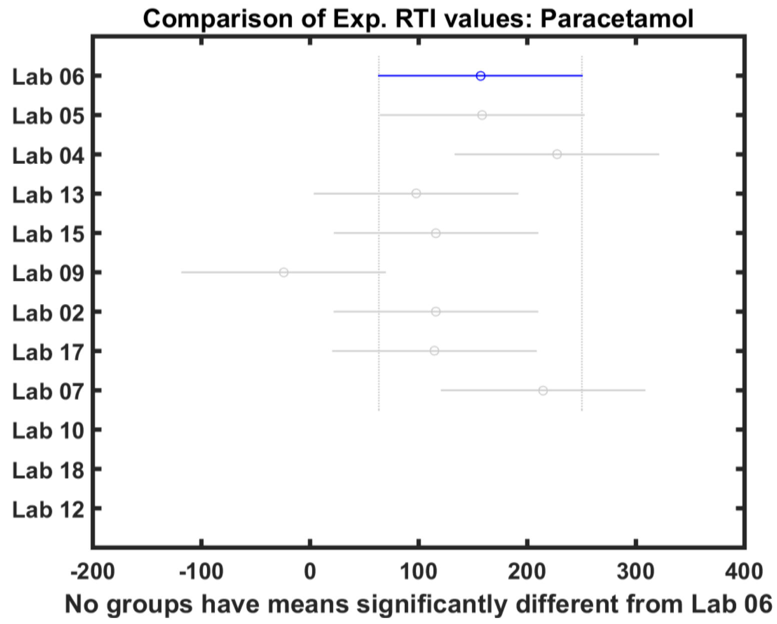

Institute	Level of identification confidence	Exp. t_R (min)	Exp. RTI	Pred. RTI	ESI mode	Application domain	MCP
Lab 6	1	3.36	156.82	216.05	+ESI	AD1	a
Lab 5	2A	4.88	158.66	216.05	+ESI	AD1	a
Lab 4	2	4.93	220.93	216.05	+ESI	AD1	a
Lab 12	N.D.	N.D.	N.D.	N.D.	N.D.	N.D.	N.D.
Lab 13	4	2.90	97.62	216.05	+ESI	AD2	a
Lab 15	1	1.50	116.02	216.05	+ESI	AD2	a
Lab 9	2	2.31	2.67	216.05	+ESI	AD3	a
Lab 2	1	2.15	115.90	216.05	+ESI	AD2	a
Lab 18	N.D.	N.D.	N.D.	N.D.	N.D.	N.D.	N.D.
Lab 17	1	2.48	114.50	216.05	+ESI	AD2	a
Lab 10	N.D.	N.D.	N.D.	N.D.	N.D.	N.D.	N.D.
Lab 7	2	7.65	214.60	216.05	+ESI	AD1	a

AD1: Exp. & Pred. tR are accepted for this candidate (box1)
AD2: Although there is error, exp. & pred. tR are accepted for this candidate (box2)
AD3: Pred./Exp. tR are not reliable (high SR; use other verification tools (box3))
N.D.: Not detected.
All institutes have acceptable exp. RTI values and the difference is **not significant at 99%CIs** of the calibration curve. **Promote "Lab 13" to level 2!**

No.	multiple comparison procedure plot	RTI details

(6)

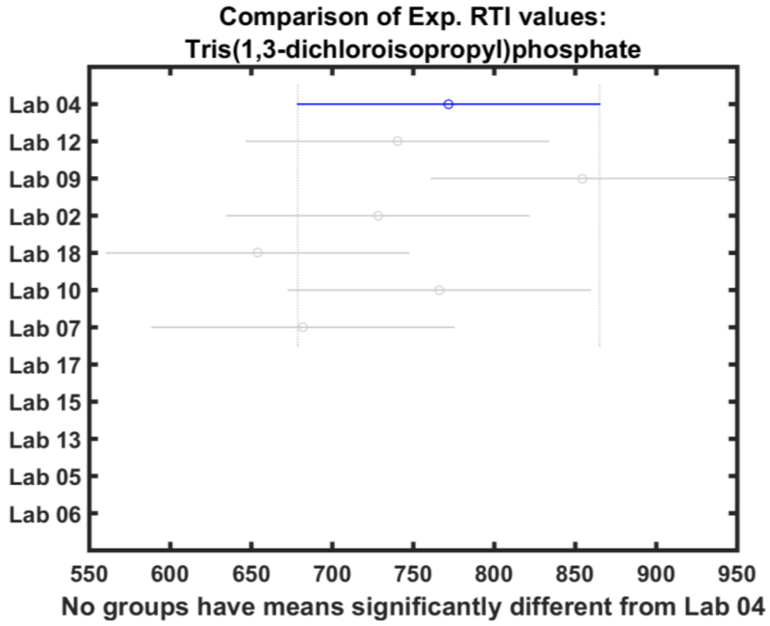

Institute	Level of identification confidence	Exp. t_R (min)	Exp. RTI	Pred. RTI	ESI mode	Application domain	MCP
Lab 6	N.D.	N.D.	N.D.	N.D.	N.D.	N.D.	N.D.
Lab 5	N.D.	N.D.	N.D.	N.D.	N.D.	N.D.	N.D.
Lab 4	2	13.44	750.73	877.93	+ESI	AD2	a
Lab 12	2a	7.18	740.14	877.93	+ESI	AD2	a
Lab 13	N.D.	N.D.	N.D.	N.D.	N.D.	N.D.	N.D.
Lab 15	N.D.	N.D.	N.D.	N.D.	N.D.	N.D.	N.D.
Lab 9	2	14.76	835.01	877.93	+ESI	AD1	a
Lab 2	1	32.35	728.14	877.93	+ESI	AD2	a
Lab 18	1	14.71	653.89	877.93	+ESI	AD3	a
Lab 17	N.D.	N.D.	N.D.	N.D.	N.D.	N.D.	N.D.
Lab 10	2	12.39	766.05	877.93	+ESI	AD2	a
Lab 7	2	16.59	681.88	877.93	+ESI	AD3	a

AD1: Exp. & Pred. tR are accepted for this candidate (box1)
AD2: Although there is error, exp. & pred. tR are accepted for this candidate (box2)
AD3: Pred./Exp. tR are not reliable (high SR; use other verification tools (box3))
N.D.: Not detected.
All institutes have acceptable exp. RTI values and the difference is **not significant at 99%CIs** of the calibration curve.

(7)

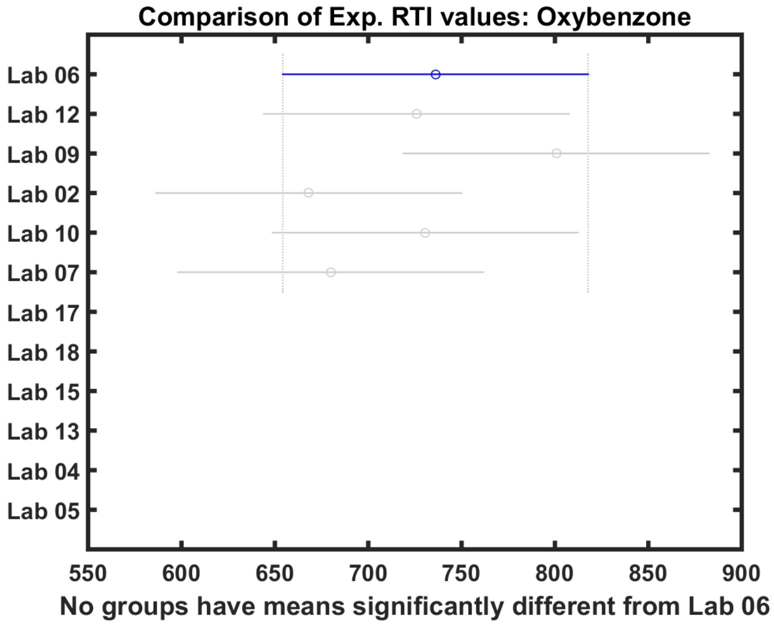

Institute	Level of identification confidence	Exp. t_R (min)	Exp. RTI	Pred. RTI	ESI mode	Application domain	MCP
Lab 6	1	10.94	735.99	641.82	+ESI	AD1	a
Lab 5	N.D.	N.D.	N.D.	N.D.	N.D.	N.D.	N.D.
Lab 4	N.D.	N.D.	N.D.	N.D.	N.D.	N.D.	N.D.
Lab 12	1	7.05	725.86	641.82	+ESI	AD1	a
Lab 13	N.D.	N.D.	N.D.	N.D.	N.D.	N.D.	N.D.
Lab 15	N.D.	N.D.	N.D.	N.D.	N.D.	N.D.	N.D.
Lab 9	2	14.00	784.13	641.82	+ESI	AD2	a
Lab 2	2	29.39	668.13	641.82	+ESI	AD1	a
Lab 18	N.D.	N.D.	N.D.	N.D.	N.D.	N.D.	N.D.
Lab 17	N.D.	N.D.	N.D.	N.D.	N.D.	N.D.	N.D.
Lab 10	1	11.83	730.58	641.82	+ESI	AD1	a
Lab 7	2	16.56	679.89	641.82	+ESI	AD1	a

AD1: Exp. & Pred. tR are accepted for this candidate (box1)
AD2: Although there is error, exp. & pred. tR are accepted for this candidate (box2)
N.D.: Not detected.
All institutes have acceptable exp. RTI values and the difference is **not significant at 99%CIs** of the calibration curve.

No.	*multiple comparison procedure plot*	*RTI details*

(8)

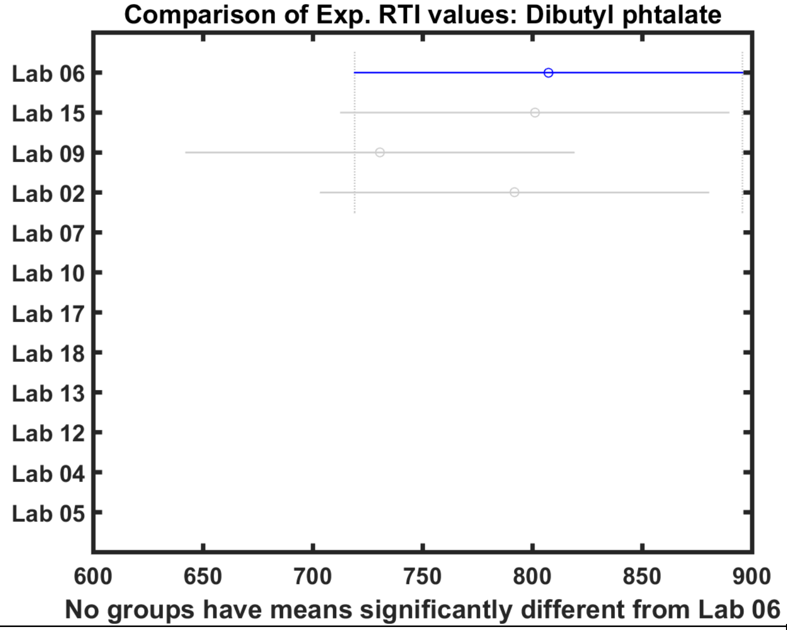

Institute	Level of identification confidence	Exp. t_R (min)	Exp. RTI	Pred. RTI	ESI mode	Application domain	MCP
Lab 6	1	11.88	807.27	737.11	+ESI	AD1	a
Lab 5	N.D.	N.D.	N.D.	N.D.	N.D.	N.D.	N.D.
Lab 4	N.D.	N.D.	N.D.	N.D.	N.D.	N.D.	N.D.
Lab 12	N.D.	N.D.	N.D.	N.D.	N.D.	N.D.	N.D.
Lab 13	N.D.	N.D.	N.D.	N.D.	N.D.	N.D.	N.D.
Lab 15	1	9.66	800.98	737.11	+ESI	AD1	a
Lab 9	2	13.01	717.68	737.11	+ESI	AD1	a
Lab 2	1	35.49	791.80	737.11	+ESI	AD1	a
Lab 18	N.D.	N.D.	N.D.	N.D.	N.D.	N.D.	N.D.
Lab 17	N.D.	N.D.	N.D.	N.D.	N.D.	N.D.	N.D.
Lab 10	N.D.	N.D.	N.D.	N.D.	N.D.	N.D.	N.D.
Lab 7	N.D.	N.D.	N.D.	N.D.	N.D.	N.D.	N.D.

AD1: Exp. & Pred. tR are accepted for this candidate (box1)
N.D.: Not detected.
All institutes have acceptable exp. RTI values and the difference is **not significant at 99%CIs** of the calibration curve.

(9)

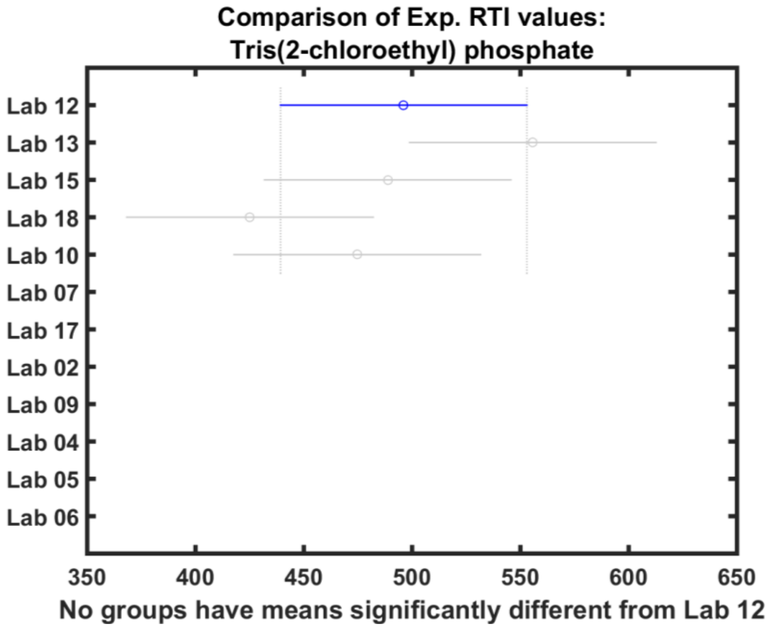

Institute	Level of identification confidence	Exp. t_R (min)	Exp. RTI	Pred. RTI	ESI mode	Application domain	MCP
Lab 6	N.D.	N.D.	N.D.	N.D.	N.D.	N.D.	N.D.
Lab 5	N.D.	N.D.	N.D.	N.D.	N.D.	N.D.	N.D.
Lab 4	N.D.	N.D.	N.D.	N.D.	N.D.	N.D.	N.D.
Lab 12	1	4.96	496.12	530.74	+ESI	AD1	a
Lab 13	2	14.89	555.72	530.74	+ESI	AD1	a
Lab 15	1	5.94	488.72	530.74	+ESI	AD1	a
Lab 9	N.D.	N.D.	N.D.	N.D.	N.D.	N.D.	N.D.
Lab 2	N.D.	N.D.	N.D.	N.D.	N.D.	N.D.	N.D.
Lab 18	1	9.71	425.15	530.74	+ESI	AD2	a
Lab 17	N.D.	N.D.	N.D.	N.D.	N.D.	N.D.	N.D.
Lab 10	2	7.79	474.69	530.74	+ESI	AD1	a
Lab 7	N.D.	N.D.	N.D.	N.D.	N.D.	N.D.	N.D.

AD1: Exp. & Pred. tR are accepted for this candidate (box1)
AD2: Although there is error, exp. & pred. tR are accepted for this candidate (box2)
N.D.: Not detected.
All institutes have acceptable exp. RTI values and the difference is **not significant at 99%CIs** of the calibration curve.

No.	*multiple comparison procedure plot*	*RTI details*

(10)

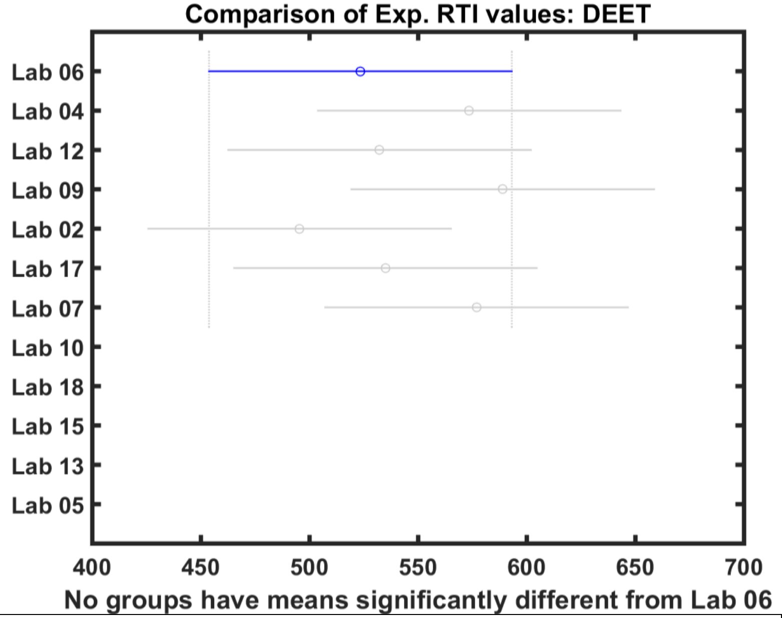

Institute	Level of identification confidence	Exp. t_R (min)	Exp. RTI	Pred. RTI	ESI mode	Application domain	MCP
Lab 6	1	8.16	523.42	516.90	+ESI	AD1	a
Lab 5	N.D.	N.D.	N.D.	N.D.	N.D.	N.D.	N.D.
Lab 4	1	10.34	557.83	516.90	+ESI	AD1	a
Lab 12	2a	5.29	532.25	516.90	+ESI	AD1	a
Lab 13	N.D.	N.D.	N.D.	N.D.	N.D.	N.D.	N.D.
Lab 15	N.D.	N.D.	N.D.	N.D.	N.D.	N.D.	N.D.
Lab 9	2	11.00	583.57	516.90	+ESI	AD1	a
Lab 2	2	20.87	495.41	516.90	+ESI	AD1	a
Lab 18	N.D.	N.D.	N.D.	N.D.	N.D.	N.D.	N.D.
Lab 17	1	9.63	534.92	516.90	+ESI	AD1	a
Lab 10	N.D.	N.D.	N.D.	N.D.	N.D.	N.D.	N.D.
Lab 7	2	14.58	576.86	516.90	+ESI	AD1	a

AD1: Exp. & Pred. tR are accepted for this candidate (box1)
N.D.: Not detected.
All institutes have acceptable exp. RTI values and the difference is **not significant at 99%CIs** of the calibration curve.

(11)

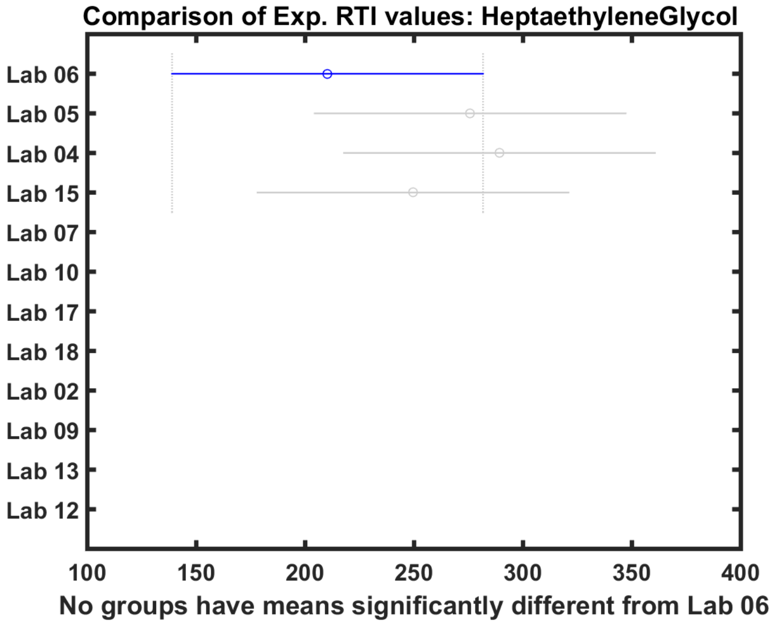

Institute	Level of identification confidence	Exp. t_R (min)	Exp. RTI	Pred. RTI	ESI mode	Application domain	MCP
Lab 6	2a	4.06	210.28	526.55	+ESI	AD4	a
Lab 5	2a	7.25	275.74	526.55	+ESI	AD3	a
Lab 4	3	5.90	281.18	526.55	+ESI	AD3	a
Lab 12	N.D.	N.D.	N.D.	N.D.	N.D.	N.D.	N.D.
Lab 13	N.D.	N.D.	N.D.	N.D.	N.D.	N.D.	N.D.
Lab 15	3	3.09	249.49	526.55	+ESI	AD3	a
Lab 9	N.D.	N.D.	N.D.	N.D.	N.D.	N.D.	N.D.
Lab 2	N.D.	N.D.	N.D.	N.D.	N.D.	N.D.	N.D.
Lab 18	N.D.	N.D.	N.D.	N.D.	N.D.	N.D.	N.D.
Lab 17	N.D.	N.D.	N.D.	N.D.	N.D.	N.D.	N.D.
Lab 10	N.D.	N.D.	N.D.	N.D.	N.D.	N.D.	N.D.
Lab 7	N.D.	N.D.	N.D.	N.D.	N.D.	N.D.	N.D.

AD3: Pred./Exp. tR are not reliable (high SR; use other verification tools (box3))
AD4: Pred./Exp. tR are not reliable (Outside the applicability domain and high SR; use other verification tools (box4))
N.D.: Not detected.
Note: This compound is outside of AD of models, but the exp. RTI is comparable. All institutes have acceptable exp. RTI values and the difference is **not significant at 99%CIs** of the calibration curve.
Promote "Lab 15" & "Lab 4" to level 2!

No.	multiple comparison procedure plot	RTI details
(12)	see below	see below

(12)

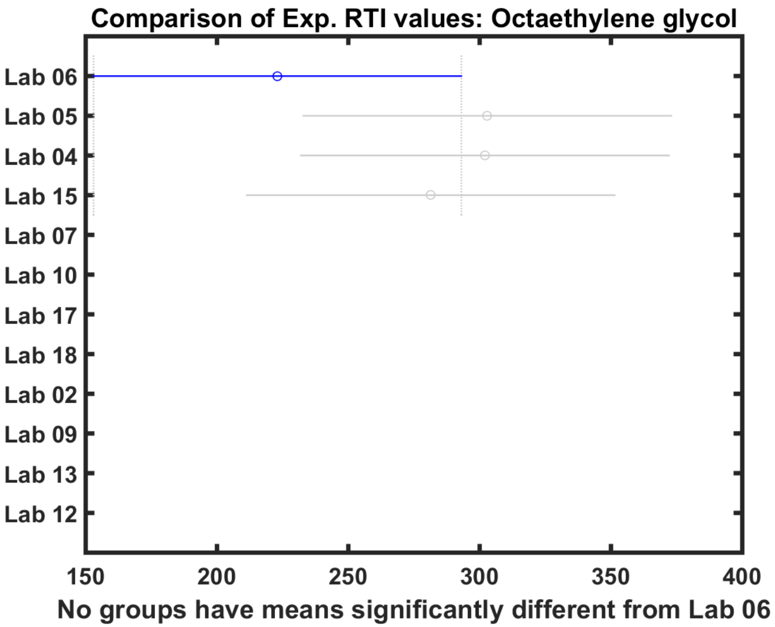

Institute	Level of identification confidence	Exp. t_R (min)	Exp. RTI	Pred. RTI	ESI mode	Application domain	MCP
Lab 6	2a	4.23	223.01	540.33	+ESI	AD4	a
Lab 5	2a	7.80	302.91	540.33	+ESI	AD3	a
Lab 4	3	6.10	293.64	540.33	+ESI	AD3	a
Lab 12	N.D.	N.D.	N.D.	N.D.	N.D.	N.D.	N.D.
Lab 13	N.D.	N.D.	N.D.	N.D.	N.D.	N.D.	N.D.
Lab 15	3	3.47	281.39	540.33	+ESI	AD3	a
Lab 9	N.D.	N.D.	N.D.	N.D.	N.D.	N.D.	N.D.
Lab 2	N.D.	N.D.	N.D.	N.D.	N.D.	N.D.	N.D.
Lab 18	N.D.	N.D.	N.D.	N.D.	N.D.	N.D.	N.D.
Lab 17	N.D.	N.D.	N.D.	N.D.	N.D.	N.D.	N.D.
Lab 10	N.D.	N.D.	N.D.	N.D.	N.D.	N.D.	N.D.
Lab 7	N.D.	N.D.	N.D.	N.D.	N.D.	N.D.	N.D.

AD3: Pred./Exp. tR are not reliable (high SR; use other verification tools (box3))
AD4: Pred./Exp. tR are not reliable (Outside the applicability domain and high SR; use other verification tools (box4))
N.D.: Not detected.
Note: This compound is outside of AD of models, but the exp. RTI is comparable. All institutes have acceptable exp. RTI values and the difference is **not significant at 99%CIs** of the calibration curve. **Promote "Lab 15" & "Lab 4" to level 2!**

(13)

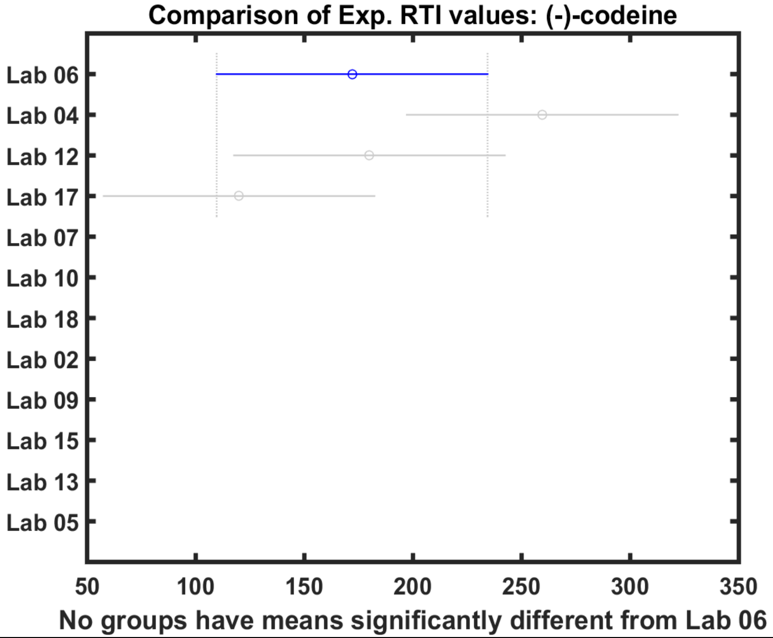

Institute	Level of identification confidence	Exp. t_R (min)	Exp. RTI	Pred. RTI	ESI mode	Application domain	MCP
Lab 6	1	3.56	172.00	163.83	+ESI	AD1	a
Lab 5	N.D.	N.D.	N.D.	N.D.	N.D.	N.D.	N.D.
Lab 4	2	5.44	252.27	163.83	+ESI	AD1	a
Lab 12	2a	2.08	179.95	163.83	+ESI	AD1	a
Lab 13	N.D.	N.D.	N.D.	N.D.	N.D.	N.D.	N.D.
Lab 15	N.D.	N.D.	N.D.	N.D.	N.D.	N.D.	N.D.
Lab 9	N.D.	N.D.	N.D.	N.D.	N.D.	N.D.	N.D.
Lab 2	N.D.	N.D.	N.D.	N.D.	N.D.	N.D.	N.D.
Lab 18	N.D.	N.D.	N.D.	N.D.	N.D.	N.D.	N.D.
Lab 17	3	2.57	119.95	163.83	+ESI	AD1	a
Lab 10	N.D.	N.D.	N.D.	N.D.	N.D.	N.D.	N.D.
Lab 7	N.D.	N.D.	N.D.	N.D.	N.D.	N.D.	N.D.

AD1: Exp. & Pred. tR are accepted for this candidate (box1)
N.D.: Not detected.
All institutes have acceptable exp. RTI values and the difference is **not significant at 99%CIs** of the calibration curve. **Promote "Lab 17" to level 2!**

No.	*multiple comparison procedure plot*	*RTI details*

(14)

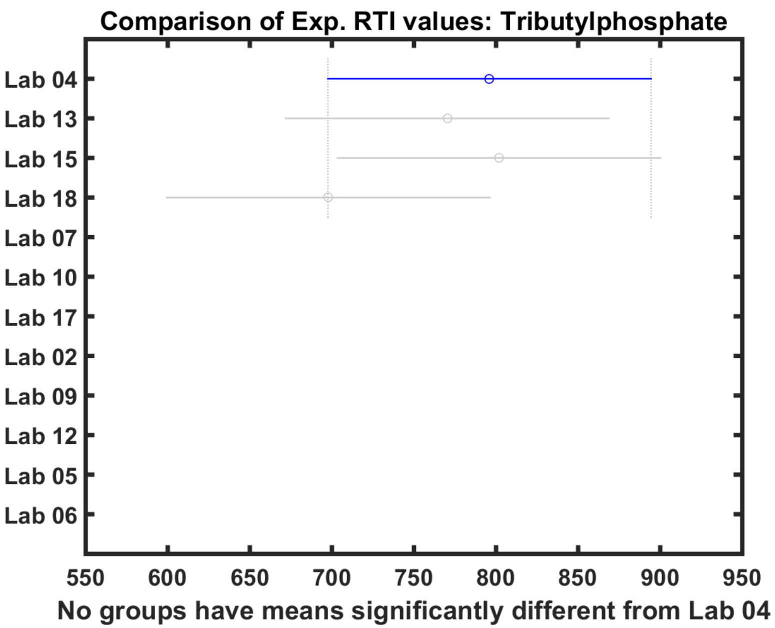

Institute	Level of identification confidence	Exp. t_R (min)	Exp. RTI	Pred. RTI	ESI mode	Application domain	MCP
Lab 6	N.D.	N.D.	N.D.	N.D.	N.D.	N.D.	N.D.
Lab 5	N.D.	N.D.	N.D.	N.D.	N.D.	N.D.	N.D.
Lab 4	1	13.81	774.28	800.96	+ESI	AD1	a
Lab 12	N.D.	N.D.	N.D.	N.D.	N.D.	N.D.	N.D.
Lab 13	3	20.51	770.25	800.96	+ESI	AD1	a
Lab 15	1	9.67	801.82	800.96	+ESI	AD1	a
Lab 9	N.D.	N.D.	N.D.	N.D.	N.D.	N.D.	N.D.
Lab 2	N.D.	N.D.	N.D.	N.D.	N.D.	N.D.	N.D.
Lab 18	1	15.67	697.81	800.96	+ESI	AD2	a
Lab 17	N.D.	N.D.	N.D.	N.D.	N.D.	N.D.	N.D.
Lab 10	N.D.	N.D.	N.D.	N.D.	N.D.	N.D.	N.D.
Lab 7	N.D.	N.D.	N.D.	N.D.	N.D.	N.D.	N.D.

AD1: Exp. & Pred. tR are accepted for this candidate (box1)
AD2: Although there is error, exp. & pred. tR are accepted for this candidate (box2)
N.D.: Not detected.
All institutes have acceptable exp. RTI values and the difference is **not significant at 99%CIs** of the calibration curve. **Promote "Lab 13" to level 2!**

(15)

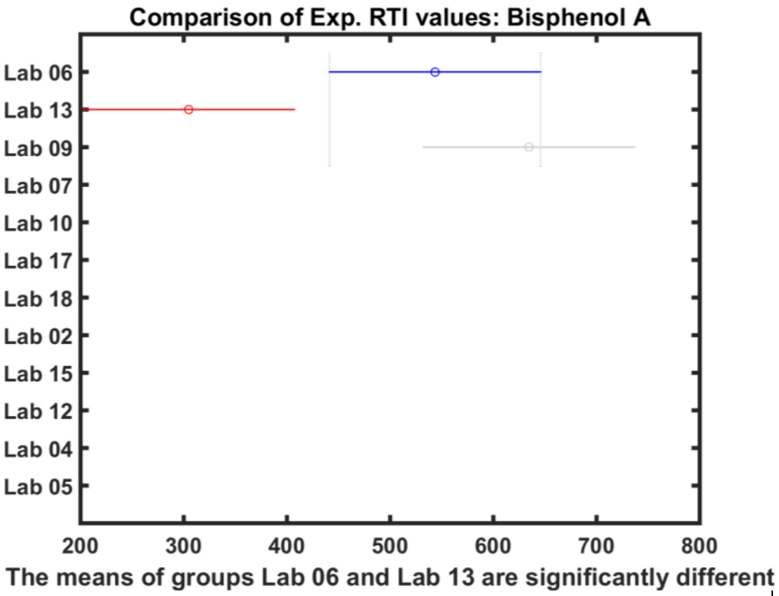

Institute	Level of identification confidence	Exp. t_R (min)	Exp. RTI	Pred. RTI	ESI mode	Application domain	MCP
Lab 6	1	8.42	543.50	679.28	+ESI	AD1	a
Lab 5	N.D.	N.D.	N.D.	N.D.	N.D.	N.D.	N.D.
Lab 4	N.D.	N.D.	N.D.	N.D.	N.D.	N.D.	N.D.
Lab 12	N.D.	N.D.	N.D.	N.D.	N.D.	N.D.	N.D.
Lab 13	4	8.33	304.97	679.28	+ESI	AD4	b
Lab 15	N.D.	N.D.	N.D.	N.D.	N.D.	N.D.	N.D.
Lab 9	1	11.65	626.76	679.28	+ESI	AD1	a
Lab 2	N.D.	N.D.	N.D.	N.D.	N.D.	N.D.	N.D.
Lab 18	N.D.	N.D.	N.D.	N.D.	N.D.	N.D.	N.D.
Lab 17	N.D.	N.D.	N.D.	N.D.	N.D.	N.D.	N.D.
Lab 10	N.D.	N.D.	N.D.	N.D.	N.D.	N.D.	N.D.
Lab 7	N.D.	N.D.	N.D.	N.D.	N.D.	N.D.	N.D.

AD1: Exp. & Pred. tR are accepted for this candidate (box1)
AD4: Pred./Exp. tR are not reliable (Outside the applicability domain and high SR; use other verification tools (box4))
N.D.: Not detected.
All institutes have acceptable exp. RTI values and the difference is **not significant at 99%CIs** of the calibration curve with the exception of "Antwerp". **Don't increase the level of identification confidence from 4 to 2 for this compound in "Lab 13" data! (It is most probably a false positive).**

No.	multiple comparison procedure plot	RTI details

(16)

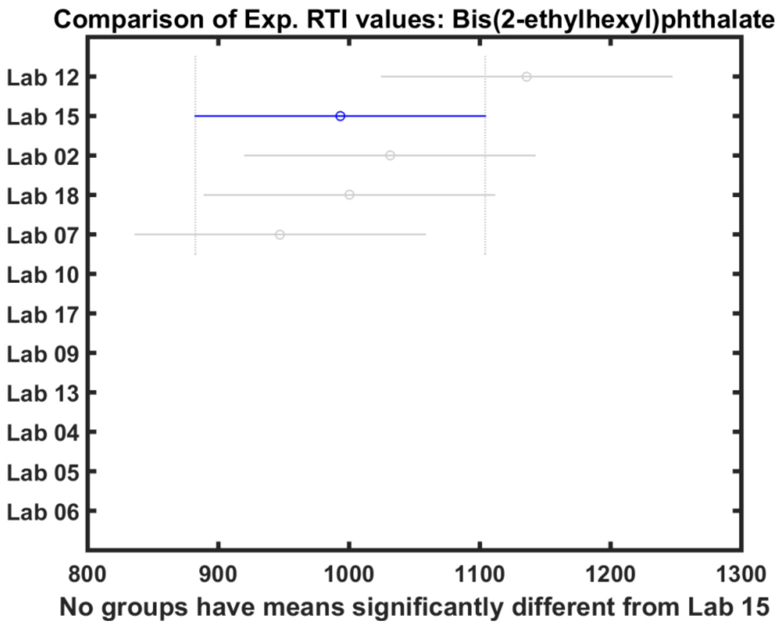

Institute	Level of identification confidence	Exp. tR	Exp. RTI	Pred. RTI	ESI mode	Application domain	MCP
Lab 6	N.D.	N.D.	N.D.	N.D.	N.D.	N.D.	N.D.
Lab 5	N.D.	N.D.	N.D.	N.D.	N.D.	N.D.	N.D.
Lab 4	N.D.	N.D.	N.D.	N.D.	N.D.	N.D.	N.D.
Lab 12	2a	10.78	1135.81	1001.82	+ESI	AD2	a
Lab 13	N.D.	N.D.	N.D.	N.D.	N.D.	N.D.	N.D.
Lab 15	1	11.95	993.21	1001.82	+ESI	AD1	a
Lab 9	N.D.	N.D.	N.D.	N.D.	N.D.	N.D.	N.D.
Lab 2	1	47.30	1031.22	1001.82	+ESI	AD1	a
Lab 18	1	22.28	1000.21	1001.82	+ESI	AD1	a
Lab 17	N.D.	N.D.	N.D.	N.D.	N.D.	N.D.	N.D.
Lab 10	N.D.	N.D.	N.D.	N.D.	N.D.	N.D.	N.D.
Lab 7	2	21.68	947.27	1001.82	+ESI	AD1	a

AD1: Exp. & Pred. tR are accepted for this candidate (box1)
AD2: Although there is error, exp. & pred. tR are accepted for this candidate (box2)
N.D.: Not detected.
All institutes have acceptable exp. RTI values and the difference is **not significant at 99%CIs** of the calibration curve.

(17)

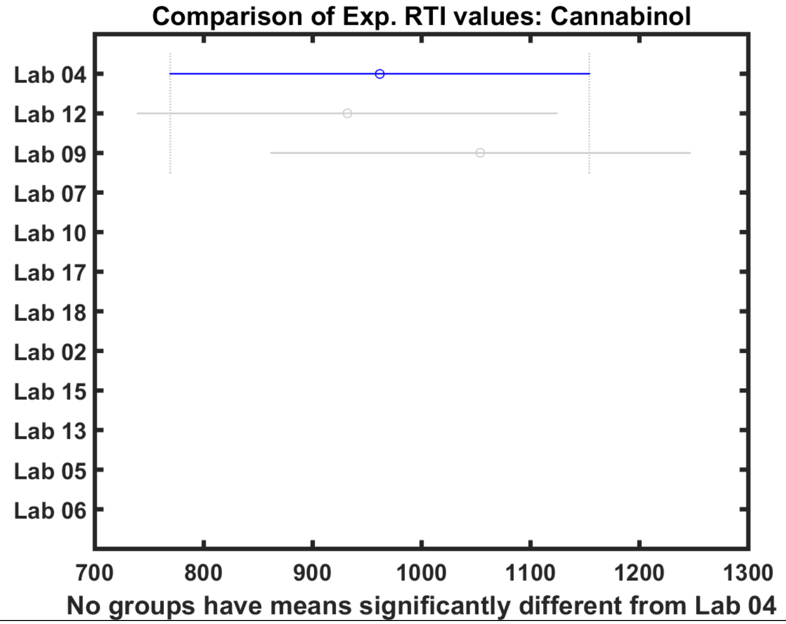

Institute	Level of identification confidence	Exp. t_R (min)	Exp. RTI	Pred. RTI	ESI mode	Application domain	MCP
Lab 6	N.D.	N.D.	N.D.	N.D.	N.D.	N.D.	N.D.
Lab 5	N.D.	N.D.	N.D.	N.D.	N.D.	N.D.	N.D.
Lab 4	2	16.40	935.41	931.59	+ESI	AD1	a
Lab 12	2a	8.92	931.66	932.79	+ESI	AD1	a
Lab 13	N.D.	N.D.	N.D.	N.D.	N.D.	N.D.	N.D.
Lab 15	N.D.	N.D.	N.D.	N.D.	N.D.	N.D.	N.D.
Lab 9	2	17.59	1024.14	932.79	+ESI	AD1	a
Lab 2	N.D.	N.D.	N.D.	N.D.	N.D.	N.D.	N.D.
Lab 18	N.D.	N.D.	N.D.	N.D.	N.D.	N.D.	N.D.
Lab 17	N.D.	N.D.	N.D.	N.D.	N.D.	N.D.	N.D.
Lab 10	N.D.	N.D.	N.D.	N.D.	N.D.	N.D.	N.D.
Lab 7	N.D.	N.D.	N.D.	N.D.	N.D.	N.D.	N.D.

AD1: Exp. & Pred. tR are accepted for this candidate (box1)
N.D.: Not detected.
All institutes have acceptable exp. RTI values and the difference is **not significant at 99%CIs** of the calibration curve.

No.	*multiple comparison procedure plot*	*RTI details*

(18)

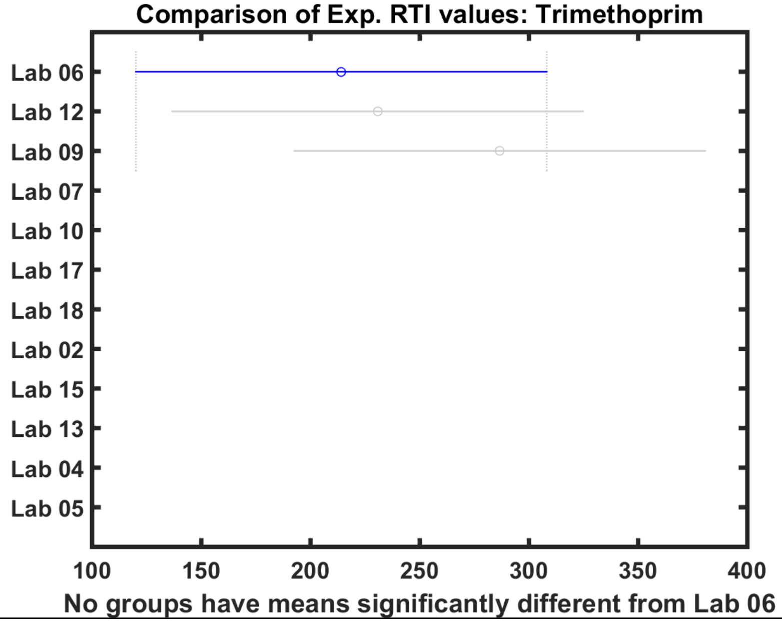

Institute	Level of identification confidence	Exp. t_R (min)	Exp. RTI	Pred. RTI	ESI mode	Application domain	MCP
Lab 6	1	4.11	214.10	276.11	+ESI	AD1	a
Lab 5	N.D.	N.D.	N.D.	N.D.	N.D.	N.D.	N.D.
Lab 4	N.D.	N.D.	N.D.	N.D.	N.D.	N.D.	N.D.
Lab 12	2a	2.54	230.69	254.91	+ESI	AD1	a
Lab 13	N.D.	N.D.	N.D.	N.D.	N.D.	N.D.	N.D.
Lab 15	N.D.	N.D.	N.D.	N.D.	N.D.	N.D.	N.D.
Lab 9	2	6.72	297.30	254.91	+ESI	AD1	a
Lab 2	N.D.	N.D.	N.D.	N.D.	N.D.	N.D.	N.D.
Lab 18	N.D.	N.D.	N.D.	N.D.	N.D.	N.D.	N.D.
Lab 17	N.D.	N.D.	N.D.	N.D.	N.D.	N.D.	N.D.
Lab 10	N.D.	N.D.	N.D.	N.D.	N.D.	N.D.	N.D.
Lab 7	N.D.	N.D.	N.D.	N.D.	N.D.	N.D.	N.D.

AD1: Exp. & Pred. tR are accepted for this candidate (box1)
N.D.: Not detected.
All institutes have acceptable exp. RTI values and the difference is **not significant at 99%CIs** of the calibration curve.

(19)

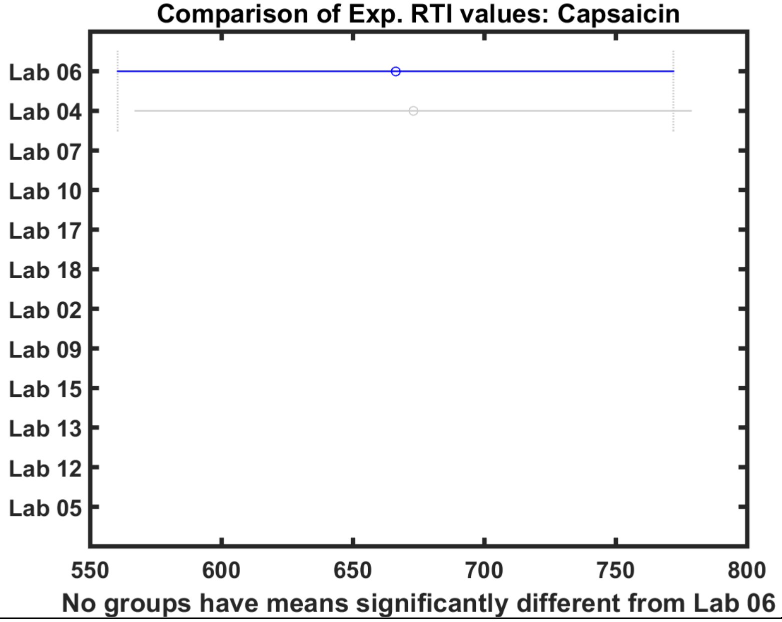

Institute	Level of identification confidence	Exp. t_R (min)	Exp. RTI	Pred. RTI	ESI mode	Application domain	MCP
Lab 6	1	10.03	666.17	655.26	+ESI	AD1	a
Lab 5	N.D.	N.D.	N.D.	N.D.	N.D.	N.D.	N.D.
Lab 4	2	11.89	654.47	655.26	+ESI	AD1	a
Lab 12	N.D.	N.D.	N.D.	N.D.	N.D.	N.D.	N.D.
Lab 13	N.D.	N.D.	N.D.	N.D.	N.D.	N.D.	N.D.
Lab 15	N.D.	N.D.	N.D.	N.D.	N.D.	N.D.	N.D.
Lab 9	N.D.	N.D.	N.D.	N.D.	N.D.	N.D.	N.D.
Lab 2	N.D.	N.D.	N.D.	N.D.	N.D.	N.D.	N.D.
Lab 18	N.D.	N.D.	N.D.	N.D.	N.D.	N.D.	N.D.
Lab 17	N.D.	N.D.	N.D.	N.D.	N.D.	N.D.	N.D.
Lab 10	N.D.	N.D.	N.D.	N.D.	N.D.	N.D.	N.D.
Lab 7	N.D.	N.D.	N.D.	N.D.	N.D.	N.D.	N.D.

AD1: Exp. & Pred. tR are accepted for this candidate (box1)
N.D.: Not detected.
All institutes have acceptable exp. RTI values and the difference is **not significant at 99%CIs** of the calibration curve.

No.	*multiple comparison procedure plot*	*RTI details*

(20)

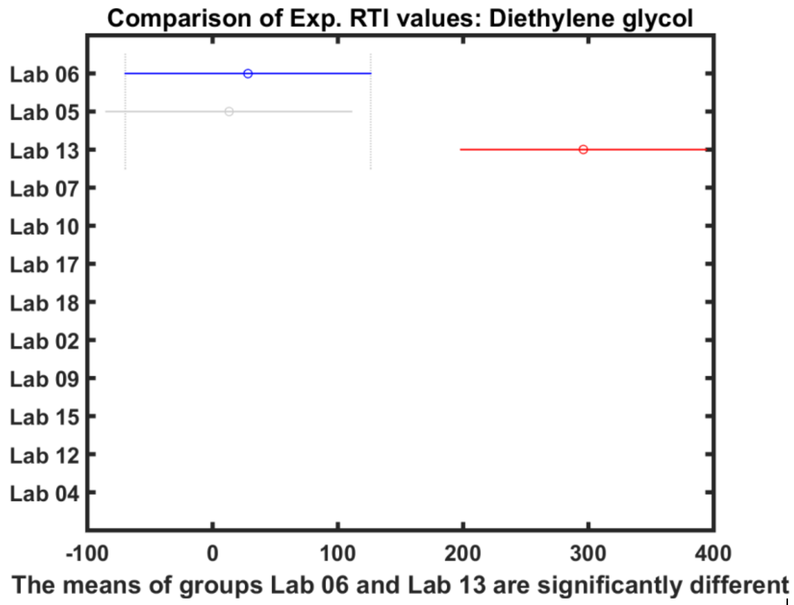

Institute	Level of identification confidence	Exp. t_R (min)	Exp. RTI	Pred. RTI	ESI mode	Application domain	MCP
Lab 6	3	1.68	28.25	111.61	+ESI	AD1	a
Lab 5	3	1.93	12.93	111.61	+ESI	AD2	a
Lab 4	N.D.	N.D.	N.D.	N.D.	N.D.	N.D.	N.D.
Lab 12	N.D.	N.D.	N.D.	N.D.	N.D.	N.D.	N.D.
Lab 13	3	8.10	296.10	111.61	+ESI	AD2	b
Lab 15	N.D.	N.D.	N.D.	N.D.	N.D.	N.D.	N.D.
Lab 9	N.D.	N.D.	N.D.	N.D.	N.D.	N.D.	N.D.
Lab 2	N.D.	N.D.	N.D.	N.D.	N.D.	N.D.	N.D.
Lab 18	N.D.	N.D.	N.D.	N.D.	N.D.	N.D.	N.D.
Lab 17	N.D.	N.D.	N.D.	N.D.	N.D.	N.D.	N.D.
Lab 10	N.D.	N.D.	N.D.	N.D.	N.D.	N.D.	N.D.
Lab 7	N.D.	N.D.	N.D.	N.D.	N.D.	N.D.	N.D.

AD1: Exp. & Pred. tR are accepted for this candidate (box1)
AD2: Although there is error, exp. & pred. tR are accepted for this candidate (box2)
N.D.: Not detected.
All institutes have acceptable exp. RTI values and the difference is **not significant at 99%CIs** of the calibration curve with the exception to “Antwerp”. **Don’t increase the level of identification confidence from 3 to 2 for this compound in “Lab 13” data! (It is most probably a false positive).**

(21)

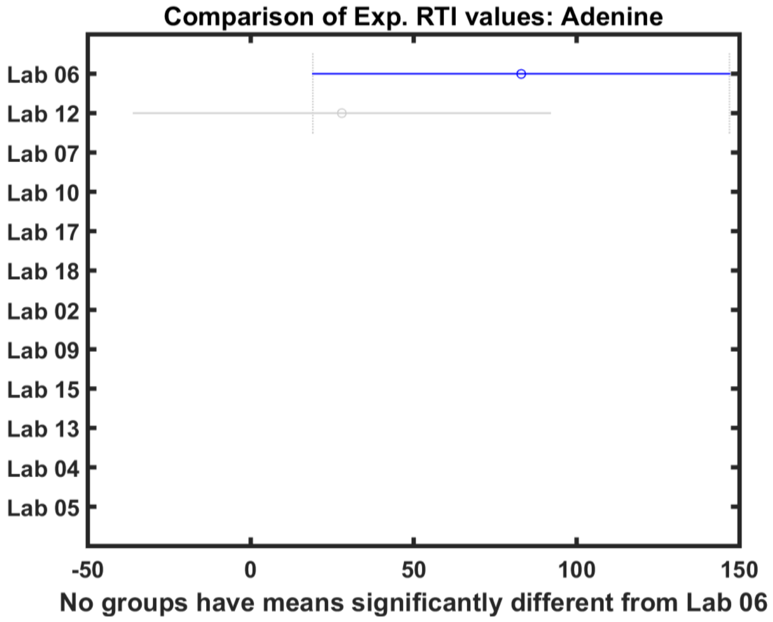

Institute	Level of identification confidence	Exp. tR	Exp. RTI	Pred. RTI	ESI mode	Application domain	MCP
Lab 6	1	2.39	82.98	126.09	+ESI	AD1	a
Lab 5	N.D.	N.D.	N.D.	N.D.	N.D.	N.D.	N.D.
Lab 4	N.D.	N.D.	N.D.	N.D.	N.D.	N.D.	N.D.
Lab 12	2a	0.69	27.96	126.09	+ESI	AD2	a
Lab 13	N.D.	N.D.	N.D.	N.D.	N.D.	N.D.	N.D.
Lab 15	N.D.	N.D.	N.D.	N.D.	N.D.	N.D.	N.D.
Lab 9	N.D.	N.D.	N.D.	N.D.	N.D.	N.D.	N.D.
Lab 2	N.D.	N.D.	N.D.	N.D.	N.D.	N.D.	N.D.
Lab 18	N.D.	N.D.	N.D.	N.D.	N.D.	N.D.	N.D.
Lab 17	N.D.	N.D.	N.D.	N.D.	N.D.	N.D.	N.D.
Lab 10	N.D.	N.D.	N.D.	N.D.	N.D.	N.D.	N.D.
Lab 7	N.D.	N.D.	N.D.	N.D.	N.D.	N.D.	N.D.

AD1: Exp. & Pred. tR are accepted for this candidate (box1)
AD2: Although there is error, exp. & pred. tR are accepted for this candidate (box2)
N.D.: Not detected.
All institutes have acceptable exp. RTI values and the difference is **not significant at 99%CIs** of the calibration curve.

No.	multiple comparison procedure plot	RTI details

(22)

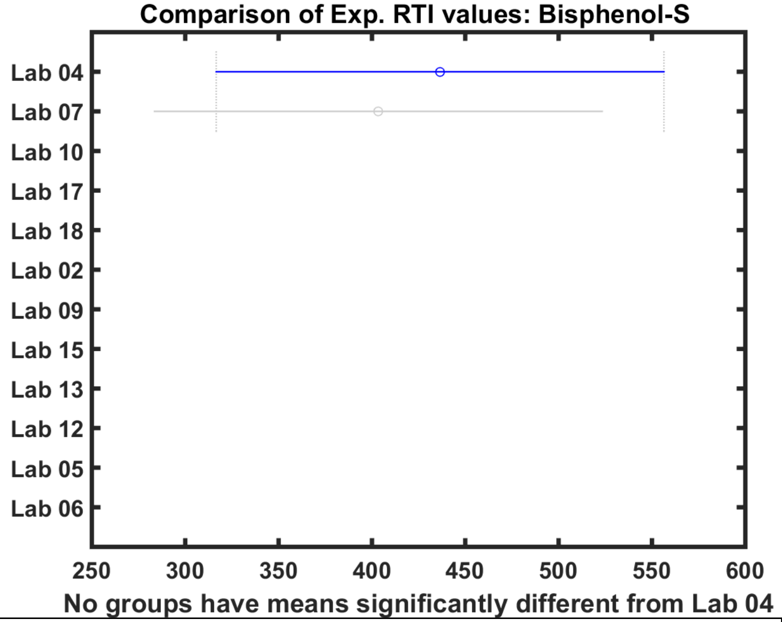

Institute	Level of identification confidence	Exp. tR	Exp. RTI	Pred. RTI	ESI mode	Application domain	MCP
Lab 6	N.D.	N.D.	N.D.	N.D.	N.D.	N.D.	N.D.
Lab 5	N.D.	N.D.	N.D.	N.D.	N.D.	N.D.	N.D.
Lab 4	1	8.20	424.49	434.99	+ESI	AD1	a
Lab 12	N.D.	N.D.	N.D.	N.D.	N.D.	N.D.	N.D.
Lab 13	N.D.	N.D.	N.D.	N.D.	N.D.	N.D.	N.D.
Lab 15	N.D.	N.D.	N.D.	N.D.	N.D.	N.D.	N.D.
Lab 9	N.D.	N.D.	N.D.	N.D.	N.D.	N.D.	N.D.
Lab 2	N.D.	N.D.	N.D.	N.D.	N.D.	N.D.	N.D.
Lab 18	N.D.	N.D.	N.D.	N.D.	N.D.	N.D.	N.D.
Lab 17	N.D.	N.D.	N.D.	N.D.	N.D.	N.D.	N.D.
Lab 10	N.D.	N.D.	N.D.	N.D.	N.D.	N.D.	N.D.
Lab 7	1	11.26	306.10	348.91	±ESI	AD1	a

AD1: Exp. & Pred. tR are accepted for this candidate (box1)
N.D.: Not detected.
All institutes have acceptable exp. RTI values and the difference is **not significant at 99%CIs** of the calibration curve.

(23)

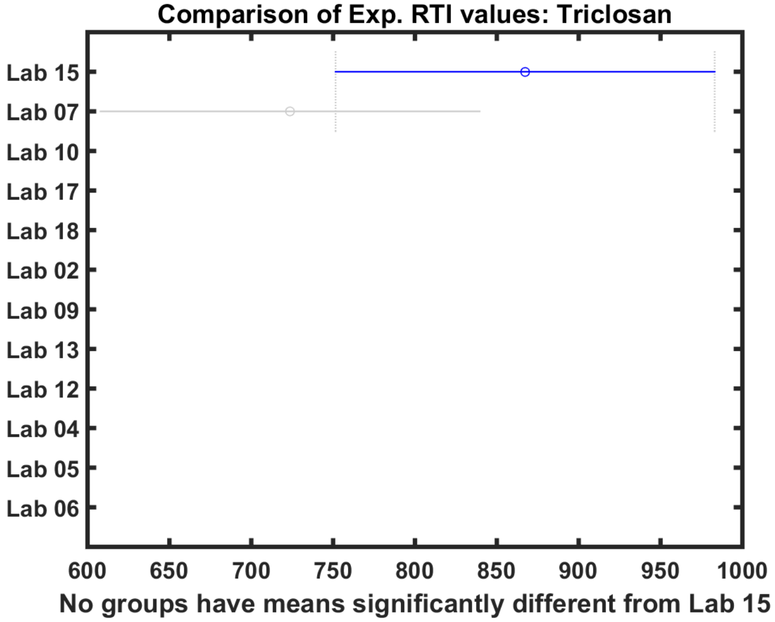

Institute	Level of identification confidence	Exp. tR	Exp. RTI	Pred. RTI	ESI mode	Application domain	MCP
Lab 6	N.D.	N.D.	N.D.	N.D.	N.D.	N.D.	N.D.
Lab 5	N.D.	N.D.	N.D.	N.D.	N.D.	N.D.	N.D.
Lab 4	N.D.	N.D.	N.D.	N.D.	N.D.	N.D.	N.D.
Lab 12	N.D.	N.D.	N.D.	N.D.	N.D.	N.D.	N.D.
Lab 13	N.D.	N.D.	N.D.	N.D.	N.D.	N.D.	N.D.
Lab 15	1	10.45	766.98	788.08	±ESI	AD1	a
Lab 9	N.D.	N.D.	N.D.	N.D.	N.D.	N.D.	N.D.
Lab 2	N.D.	N.D.	N.D.	N.D.	N.D.	N.D.	N.D.
Lab 18	N.D.	N.D.	N.D.	N.D.	N.D.	N.D.	N.D.
Lab 17	N.D.	N.D.	N.D.	N.D.	N.D.	N.D.	N.D.
Lab 10	N.D.	N.D.	N.D.	N.D.	N.D.	N.D.	N.D.
Lab 7	2	17.40	581.51	788.08	±ESI	AD3	a

AD1: Exp. & Pred. tR are accepted for this candidate (box1)
AD3: Pred./Exp. tR are not reliable (high SR; use other verification tools (box3))
N.D.: Not detected.
Note: All institutes have acceptable exp. RTI values and the difference is **not significant at 99%CIs** of the calibration curve.

No.	*multiple comparison procedure plot*	*RTI details*

(24)

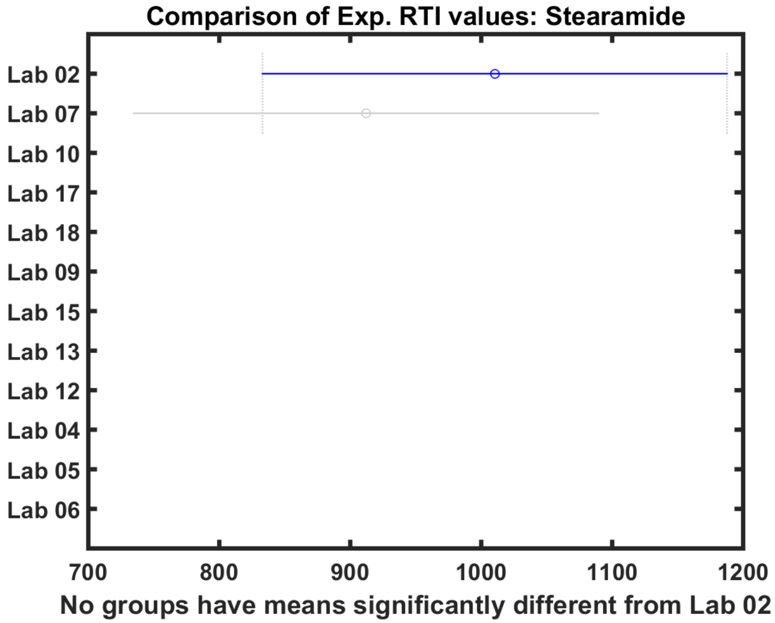

Institute	Level of identification confidence	Exp. tR	Exp. RTI	Pred. RTI	ESI mode	Application domain	MCP
Lab 6	N.D.	N.D.	N.D.	N.D.	N.D.	N.D.	N.D.
Lab 5	N.D.	N.D.	N.D.	N.D.	N.D.	N.D.	N.D.
Lab 4	N.D.	N.D.	N.D.	N.D.	N.D.	N.D.	N.D.
Lab 12	N.D.	N.D.	N.D.	N.D.	N.D.	N.D.	N.D.
Lab 13	N.D.	N.D.	N.D.	N.D.	N.D.	N.D.	N.D.
Lab 15	N.D.	N.D.	N.D.	N.D.	N.D.	N.D.	N.D.
Lab 9	N.D.	N.D.	N.D.	N.D.	N.D.	N.D.	N.D.
Lab 2	2	46.27	1010.34	882.75	+ESI	AD2	a
Lab 18	N.D.	N.D.	N.D.	N.D.	N.D.	N.D.	N.D.
Lab 17	N.D.	N.D.	N.D.	N.D.	N.D.	N.D.	N.D.
Lab 10	N.D.	N.D.	N.D.	N.D.	N.D.	N.D.	N.D.
Lab 7	2	21.00	912.07	882.75	+ESI	AD1	a

AD1: Exp. & Pred. tR are accepted for this candidate (box1)
AD2: Although there is error, exp. & pred. tR are accepted for this candidate (box2)
N.D.: Not detected.
All institutes have acceptable exp. RTI values and the difference is **not significant at 99%CIs** of the calibration curve.

(25)

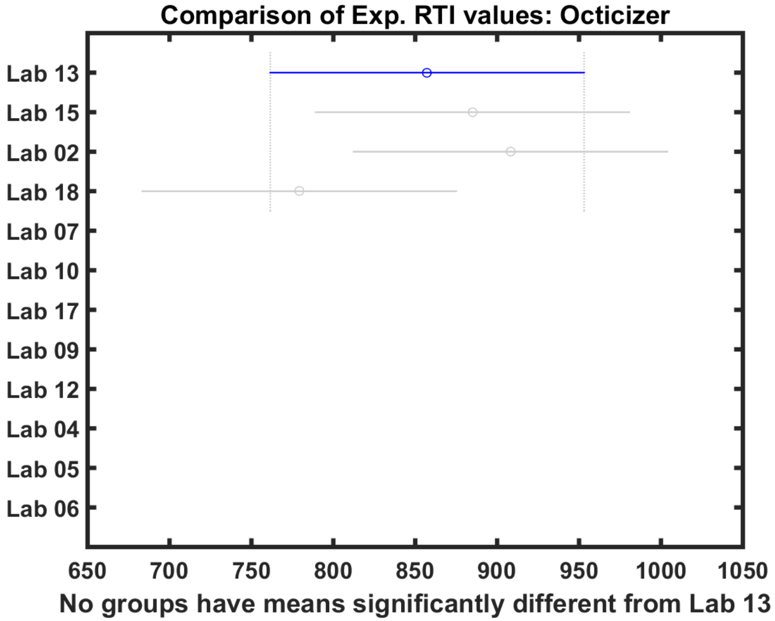

Institute	Level of identification confidence	Exp. tR	Exp. RTI	Pred. RTI	ESI mode	Application domain	MCP
Lab 6	N.D.	N.D.	N.D.	N.D.	N.D.	N.D.	N.D.
Lab 5	N.D.	N.D.	N.D.	N.D.	N.D.	N.D.	N.D.
Lab 4	N.D.	N.D.	N.D.	N.D.	N.D.	N.D.	N.D.
Lab 12	N.D.	N.D.	N.D.	N.D.	N.D.	N.D.	N.D.
Lab 13	3	22.78	857.28	890.59	+ESI	AD1	a
Lab 15	1	10.66	884.93	890.59	+ESI	AD1	a
Lab 9	N.D.	N.D.	N.D.	N.D.	N.D.	N.D.	N.D.
Lab 2	3	41.23	908.16	890.59	+ESI	AD1	a
Lab 18	1	17.45	779.24	890.59	+ESI	AD2	a
Lab 17	N.D.	N.D.	N.D.	N.D.	N.D.	N.D.	N.D.
Lab 10	N.D.	N.D.	N.D.	N.D.	N.D.	N.D.	N.D.
Lab 7	N.D.	N.D.	N.D.	N.D.	N.D.	N.D.	N.D.

AD1: Exp. & Pred. tR are accepted for this candidate (box1)
AD2: Although there is error, exp. & pred. tR are accepted for this candidate (box2)
N.D.: Not detected.
All institutes have acceptable exp. RTI values and the difference is **not significant at 99%CIs** of the calibration curve. **Promote "Lab 13" & "Lab 2" to level 2!**

No.	*multiple comparison procedure plot*	*RTI details*

(26)

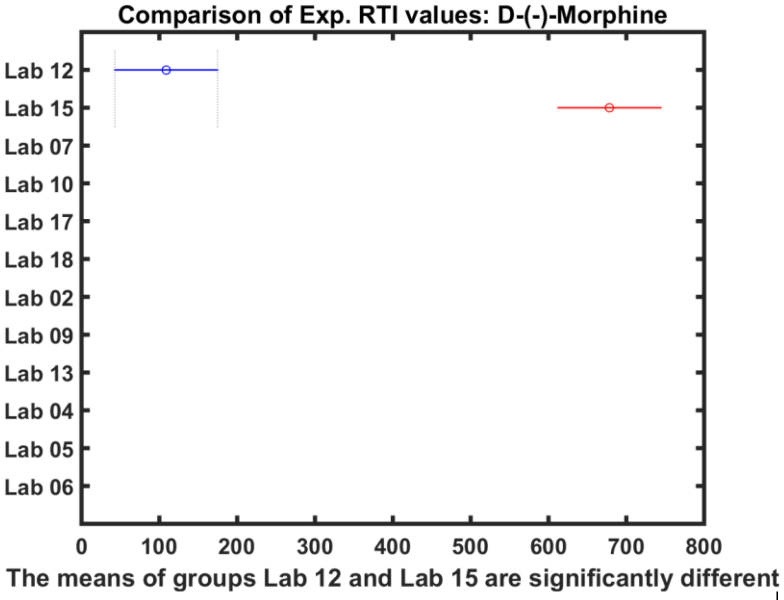

Institute	Level of identification confidence	Exp. tR	Exp. RTI	Pred. RTI	ESI mode	Application domain	MCP
Lab 6	N.D.	N.D.	N.D.	N.D.	N.D.	N.D.	N.D.
Lab 5	N.D.	N.D.	N.D.	N.D.	N.D.	N.D.	N.D.
Lab 4	N.D.	N.D.	N.D.	N.D.	N.D.	N.D.	N.D.
Lab 12	2a	1.43	108.79	111.19	+ESI	AD1	a
Lab 13	N.D.	N.D.	N.D.	N.D.	N.D.	N.D.	N.D.
Lab 15	4	8.20	678.43	111.19	+ESI	AD4a	b
Lab 9	N.D.	N.D.	N.D.	N.D.	N.D.	N.D.	N.D.
Lab 2	N.D.	N.D.	N.D.	N.D.	N.D.	N.D.	N.D.
Lab 18	N.D.	N.D.	N.D.	N.D.	N.D.	N.D.	N.D.
Lab 17	N.D.	N.D.	N.D.	N.D.	N.D.	N.D.	N.D.
Lab 10	N.D.	N.D.	N.D.	N.D.	N.D.	N.D.	N.D.
Lab 7	N.D.	N.D.	N.D.	N.D.	N.D.	N.D.	N.D.

AD1: Exp. & Pred. tR are accepted for this candidate (box1)
AD4a: Pred./Exp. tR are not reliable (high SR; the LC method used is not well suited for this compound, it could be a false positive) (box4)
N.D.: Not detected.
Note: This compound is false positive in "**Lab 15**" data, as the experimental and predicted RTI do not match comparing to "**Lab 12**" data which is at level of identification 2a.

(27)

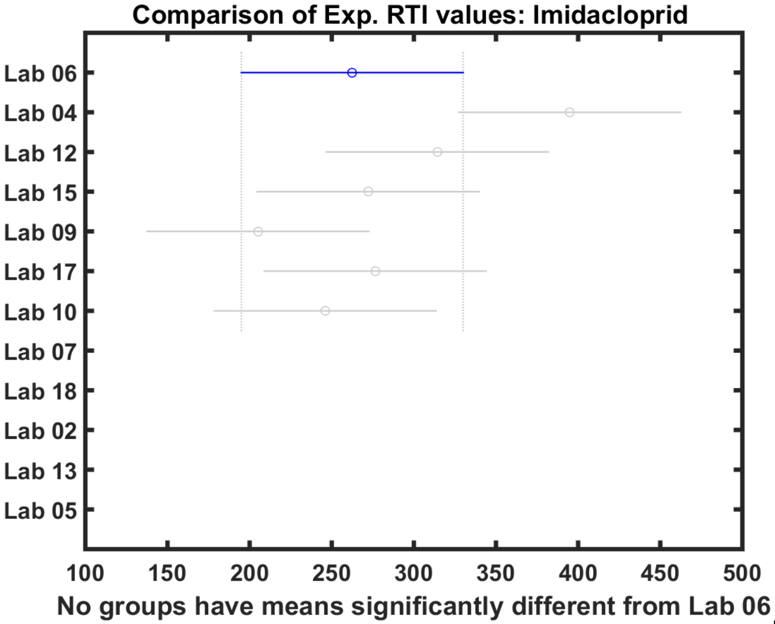

Institute	Level of identification confidence	Exp. tR	Exp. RTI	Pred. RTI	ESI mode	Application domain	MCP
Lab 6	1	4.74	262.47	248.89	+ESI	AD1	a
Lab 5	N.D.	N.D.	N.D.	N.D.	N.D.	N.D.	N.D.
Lab 4	1	7.55	383.99	248.89	+ESI	AD2	a
Lab 12	2a	3.30	314.37	248.89	+ESI	AD1	a
Lab 13	N.D.	N.D.	N.D.	N.D.	N.D.	N.D.	N.D.
Lab 15	4	3.36	272.15	248.89	+ESI	AD1	a
Lab 9	2	5.56	220.01	248.89	+ESI	AD1	a
Lab 2	N.D.	N.D.	N.D.	N.D.	N.D.	N.D.	N.D.
Lab 18	N.D.	N.D.	N.D.	N.D.	N.D.	N.D.	N.D.
Lab 17	3	5.23	276.40	248.89	+ESI	AD1	a
Lab 10	1	4.18	246.04	248.89	+ESI	AD1	a
Lab 7	N.D.	N.D.	N.D.	N.D.	N.D.	N.D.	N.D.

AD1: Exp. & Pred. tR are accepted for this candidate (box1)
AD2: Although there is error, exp. & pred. tR are accepted for this candidate (box2)
N.D.: Not detected.
All institutes have acceptable exp. RTI values and the difference is **not significant at 99%CIs** of the calibration curve. **Promote "Lab 15" & "Lab 17" to level 2!**

No.	multiple comparison procedure plot	RTI details

(28)

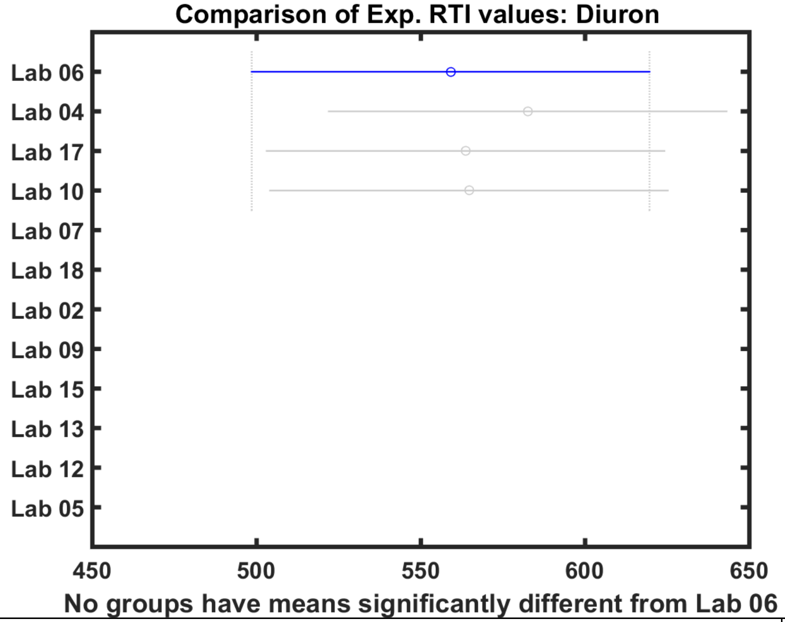

Institute	Level of identification confidence	Exp. tR	Exp. RTI	Pred. RTI	ESI mode	Application domain	MCP
Lab 6	1	8.63	559.05	550.53	+ESI	AD1	a
Lab 5	N.D.	N.D.	N.D.	N.D.	N.D.	N.D.	N.D.
Lab 4	1	10.48	566.55	550.53	+ESI	AD1	a
Lab 12	N.D.	N.D.	N.D.	N.D.	N.D.	N.D.	N.D.
Lab 13	N.D.	N.D.	N.D.	N.D.	N.D.	N.D.	N.D.
Lab 15	N.D.	N.D.	N.D.	N.D.	N.D.	N.D.	N.D.
Lab 9	N.D.	N.D.	N.D.	N.D.	N.D.	N.D.	N.D.
Lab 2	N.D.	N.D.	N.D.	N.D.	N.D.	N.D.	N.D.
Lab 18	N.D.	N.D.	N.D.	N.D.	N.D.	N.D.	N.D.
Lab 17	1	10.11	563.60	570.79	+ESI	AD1	a
Lab 10	1	9.21	564.63	550.53	+ESI	AD1	a
Lab 7	N.D.	N.D.	N.D.	N.D.	N.D.	N.D.	N.D.

AD1: Exp. & Pred. tR are accepted for this candidate (box1)
N.D.: Not detected.
All institutes have acceptable exp. RTI values and the difference is **not significant at 99%CIs** of the calibration curve.

(29)

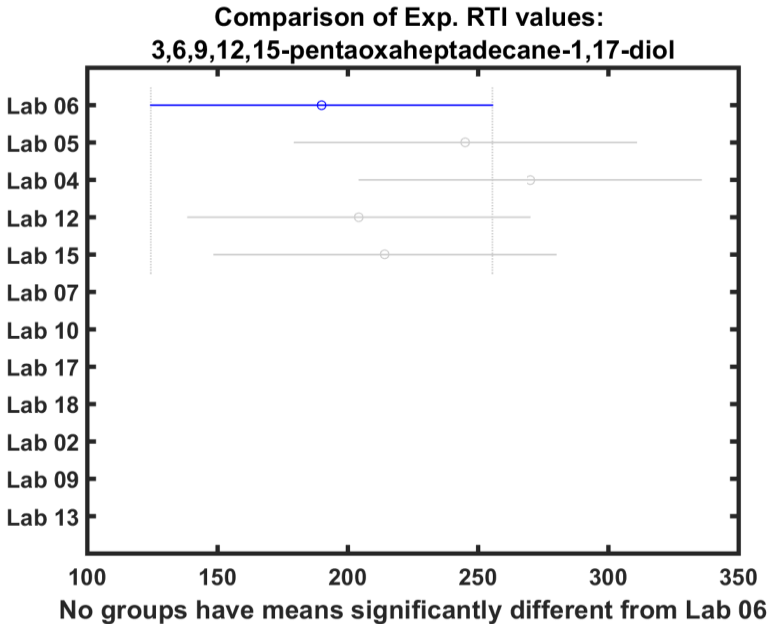

Institute	Level of identification confidence	Exp. tR	Exp. RTI	Pred. RTI	ESI mode	Application domain	MCP
Lab 6	2a	3.79	189.91	498.74	+ESI	AD4a	a
Lab 5	2a	6.63	245.11	498.74	+ESI	AD3	a
Lab 4	3	5.60	262.49	498.74	+ESI	AD3	a
Lab 12	2a	2.30	204.22	498.74	+ESI	AD4a	a
Lab 13	N.D.	N.D.	N.D.	N.D.	N.D.	N.D.	N.D.
Lab 15	3	2.67	214.23	498.74	+ESI	AD4a	a
Lab 9	N.D.	N.D.	N.D.	N.D.	N.D.	N.D.	N.D.
Lab 2	N.D.	N.D.	N.D.	N.D.	N.D.	N.D.	N.D.
Lab 18	N.D.	N.D.	N.D.	N.D.	N.D.	N.D.	N.D.
Lab 17	N.D.	N.D.	N.D.	N.D.	N.D.	N.D.	N.D.
Lab 10	N.D.	N.D.	N.D.	N.D.	N.D.	N.D.	N.D.
Lab 7	N.D.	N.D.	N.D.	N.D.	N.D.	N.D.	N.D.

AD3: Pred./Exp. tR are not reliable (high SR; use other verification tools (box3))
AD4a: Pred./Exp. tR are not reliable (Outside the applicability domain and high SR; use other verification tools (box4))
N.D.: Not detected.
Note: This compound is outside of AD of models, but the exp. RTI is comparable. All institutes have acceptable exp. RTI values and the difference is **not significant at 99%CIs** of the calibration curve.
Promote "Lab 15" & "Lab 4" to level 2!

No.	*multiple comparison procedure plot*	*RTI details*

(30)

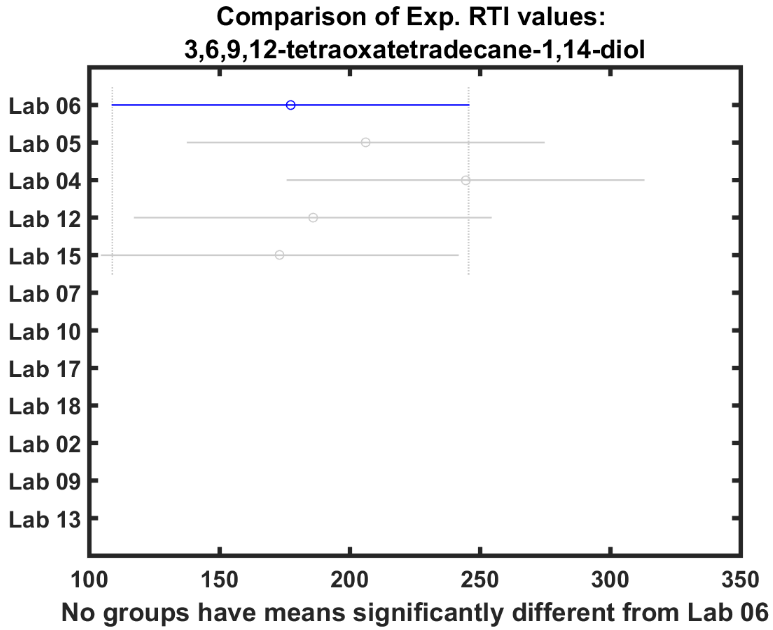

Institute	Level of identification confidence	Exp. tR	Exp. RTI	Pred. RTI	ESI mode	Application domain	MCP
Lab 6	2a	3.63	177.18	441.46	+ESI	AD3	a
Lab 5	2a	5.84	206.08	441.46	+ESI	AD3	a
Lab 4	3	5.20	237.57	441.46	+ESI	AD3	a
Lab 12	2a	2.13	185.77	441.46	+ESI	AD3	a
Lab 13	N.D.	N.D.	N.D.	N.D.	N.D.	N.D.	N.D.
Lab 15	3	2.18	173.10	441.46	+ESI	AD3	a
Lab 9	N.D.	N.D.	N.D.	N.D.	N.D.	N.D.	N.D.
Lab 2	N.D.	N.D.	N.D.	N.D.	N.D.	N.D.	N.D.
Lab 18	N.D.	N.D.	N.D.	N.D.	N.D.	N.D.	N.D.
Lab 17	N.D.	N.D.	N.D.	N.D.	N.D.	N.D.	N.D.
Lab 10	N.D.	N.D.	N.D.	N.D.	N.D.	N.D.	N.D.
Lab 7	N.D.	N.D.	N.D.	N.D.	N.D.	N.D.	N.D.

AD3: Pred./Exp. tR are not reliable (high SR; use other verification tools (box3))
N.D.: Not detected.
Note: This compound is outside of AD of models, but the exp. RTI is comparable. All institutes have acceptable exp. RTI values and the difference is **not significant at 99%CIs** of the calibration curve. **Promote "Lab 15" & "Lab 4" to level 2!**

(31)

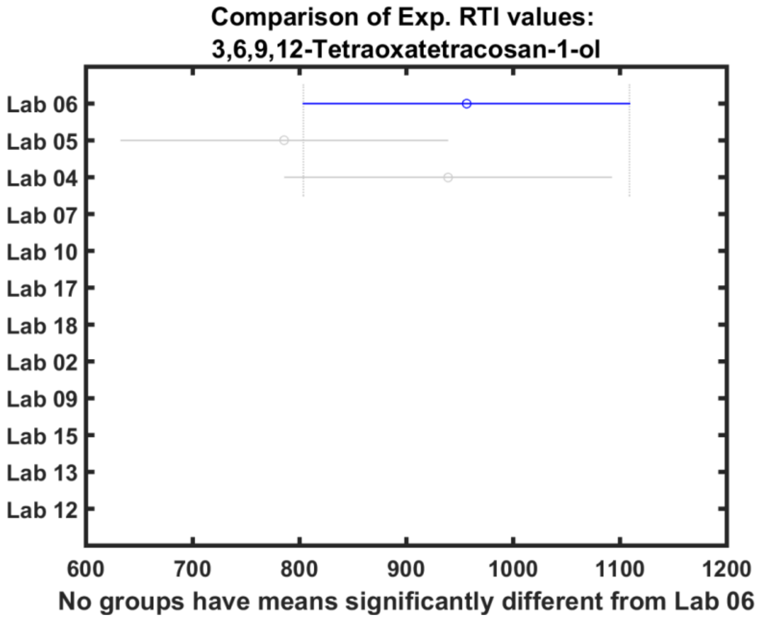

Institute	Level of identification confidence	Exp. tR	Exp. RTI	Pred. RTI	ESI mode	Application domain	MCP
Lab 6	1	13.83	956.20	806.31	+ESI	AD2	a
Lab 5	3	17.57	785.69	806.31	+ESI	AD1	a
Lab 4	3	16.05	913.48	806.31	+ESI	AD2	a
Lab 12	N.D.	N.D.	N.D.	N.D.	N.D.	N.D.	N.D.
Lab 13	N.D.	N.D.	N.D.	N.D.	N.D.	N.D.	N.D.
Lab 15	N.D.	N.D.	N.D.	N.D.	N.D.	N.D.	N.D.
Lab 9	N.D.	N.D.	N.D.	N.D.	N.D.	N.D.	N.D.
Lab 2	N.D.	N.D.	N.D.	N.D.	N.D.	N.D.	N.D.
Lab 18	N.D.	N.D.	N.D.	N.D.	N.D.	N.D.	N.D.
Lab 17	N.D.	N.D.	N.D.	N.D.	N.D.	N.D.	N.D.
Lab 10	N.D.	N.D.	N.D.	N.D.	N.D.	N.D.	N.D.
Lab 7	N.D.	N.D.	N.D.	N.D.	N.D.	N.D.	N.D.

AD1: Exp. & Pred. tR are accepted for this candidate (box1)
AD2: Although there is error, exp. & pred. tR are accepted for this candidate (box2)
N.D.: Not detected.
All institutes have acceptable exp. RTI values and the difference is **not significant at 99%CIs** of the calibration curve. **Promote "Lab 5" & "Lab 4" to level 2!**

No.	*multiple comparison procedure plot*	*RTI details*

(32)

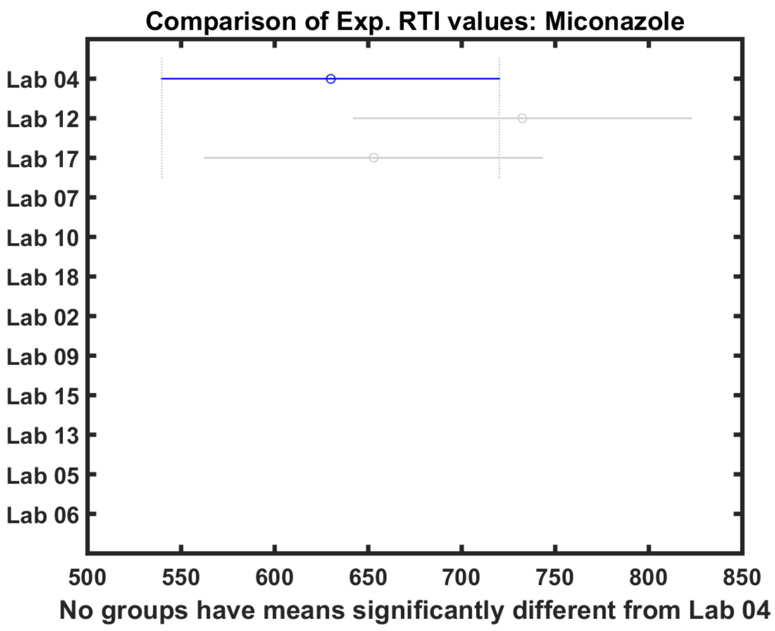

Institute	Level of identification confidence	Exp. tR	Exp. RTI	Pred. RTI	ESI mode	Application domain	MCP
Lab 6	N.D.	N.D.	N.D.	N.D.	N.D.	N.D.	N.D.
Lab 5	N.D.	N.D.	N.D.	N.D.	N.D.	N.D.	N.D.
Lab 4	2	11.22	612.66	841.19	+ESI	AD3	a
Lab 12	2a	7.11	732.56	841.19	+ESI	AD2	a
Lab 13	N.D.	N.D.	N.D.	N.D.	N.D.	N.D.	N.D.
Lab 15	N.D.	N.D.	N.D.	N.D.	N.D.	N.D.	N.D.
Lab 9	N.D.	N.D.	N.D.	N.D.	N.D.	N.D.	N.D.
Lab 2	N.D.	N.D.	N.D.	N.D.	N.D.	N.D.	N.D.
Lab 18	N.D.	N.D.	N.D.	N.D.	N.D.	N.D.	N.D.
Lab 17	3	11.63	652.83	841.19	+ESI	AD2	a
Lab 10	N.D.	N.D.	N.D.	N.D.	N.D.	N.D.	N.D.
Lab 7	N.D.	N.D.	N.D.	N.D.	N.D.	N.D.	N.D.

AD2: Although there is error, exp. & pred. tR are accepted for this candidate (box2)
AD3: Pred./Exp. tR are not reliable (high SR; use other verification tools (box3))
N.D.: Not detected.
All institutes have acceptable exp. RTI values and the difference is **not significant at 99%CIs** of the calibration curve. **Promote "Lab 17" to level 2!**

(33)

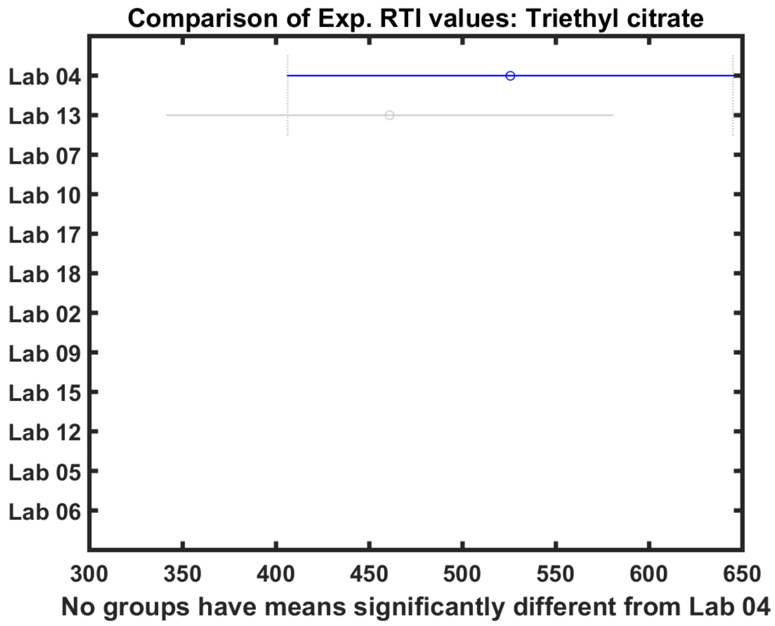

Institute	Level of identification confidence	Exp. tR	Exp. RTI	Pred. RTI	ESI mode	Application domain	MCP
Lab 6	N.D.	N.D.	N.D.	N.D.	N.D.	N.D.	N.D.
Lab 5	N.D.	N.D.	N.D.	N.D.	N.D.	N.D.	N.D.
Lab 4	2	9.59	511.16	378.61	+ESI	AD2	a
Lab 12	N.D.	N.D.	N.D.	N.D.	N.D.	N.D.	N.D.
Lab 13	4	12.41	460.96	378.61	+ESI	AD1	a
Lab 15	N.D.	N.D.	N.D.	N.D.	N.D.	N.D.	N.D.
Lab 9	N.D.	N.D.	N.D.	N.D.	N.D.	N.D.	N.D.
Lab 2	N.D.	N.D.	N.D.	N.D.	N.D.	N.D.	N.D.
Lab 18	N.D.	N.D.	N.D.	N.D.	N.D.	N.D.	N.D.
Lab 17	N.D.	N.D.	N.D.	N.D.	N.D.	N.D.	N.D.
Lab 10	N.D.	N.D.	N.D.	N.D.	N.D.	N.D.	N.D.
Lab 7	N.D.	N.D.	N.D.	N.D.	N.D.	N.D.	N.D.

AD1: Exp. & Pred. tR are accepted for this candidate (box1)
AD2: Although there is error, exp. & pred. tR are accepted for this candidate (box2)
N.D.: Not detected.
All institutes have acceptable exp. RTI values and the difference is **not significant at 99%CIs** of the calibration curve. **Promote "Lab 13" to level 2!**

No.	*multiple comparison procedure plot*	*RTI details*
(34)		

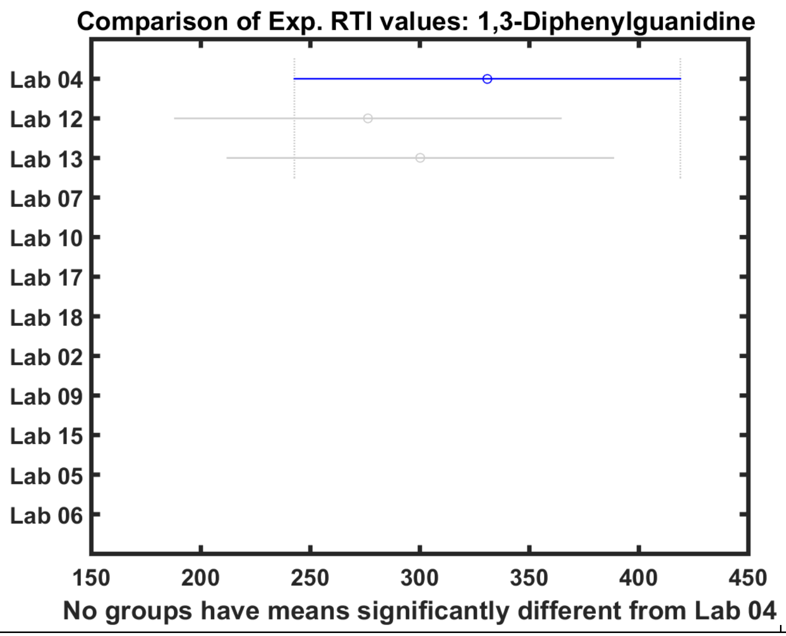

Institute	Level of identification confidence	Exp. tR	Exp. RTI	Pred. RTI	ESI mode	Application domain	MCP
Lab 6	N.D.	N.D.	N.D.	N.D.	N.D.	N.D.	N.D.
Lab 5	N.D.	N.D.	N.D.	N.D.	N.D.	N.D.	N.D.
Lab 4	1	6.55	321.68	361.26	+ESI	AD1	a
Lab 12	2a	2.96	276.26	361.26	+ESI	AD1	a
Lab 13	3	8.20	300.23	361.26	+ESI	AD1	a
Lab 15	N.D.	N.D.	N.D.	N.D.	N.D.	N.D.	N.D.
Lab 9	N.D.	N.D.	N.D.	N.D.	N.D.	N.D.	N.D.
Lab 2	N.D.	N.D.	N.D.	N.D.	N.D.	N.D.	N.D.
Lab 18	N.D.	N.D.	N.D.	N.D.	N.D.	N.D.	N.D.
Lab 17	N.D.	N.D.	N.D.	N.D.	N.D.	N.D.	N.D.
Lab 10	N.D.	N.D.	N.D.	N.D.	N.D.	N.D.	N.D.
Lab 7	N.D.	N.D.	N.D.	N.D.	N.D.	N.D.	N.D.

AD1: Exp. & Pred. tR are accepted for this candidate (box1)
AD2: Although there is error, exp. & pred. tR are accepted for this candidate (box2)
N.D.: Not detected.
All institutes have acceptable exp. RTI values and the difference is **not significant at 99%CIs** of the calibration curve. **Promote “Lab 13” to level 2!**

Table S7 Practical application of RTI and FOR-IDENT platform for identification of emerging pollutants. Compounds removed from the list as likely wrongly identified

Name	InChi key	CAS	Δ logD
pipamperone	AXKPFOAXAHJUAG-UHFFFAOYSA-N	1893-33-0	8.98
etamiphyllin	AWKLBIOQCIORSB-UHFFFAOYSA-N	314-35-2	6.47
fleroxacin	XBJBPGROQZJDOJ-UHFFFAOYSA-N	79660-72-3	6.14
Dipropetryn	NPWMZOGDXOFZIN-UHFFFAOYSA-N	4147-51-7	5.94
morphine	BQJCRHHNABKAKU-KBQPJGBKSA-N	57-27-2	5.39
norcodeine	HKOIXWVRNLGFOR-YANNOFPNSA-N	467-15-2	5.37
galantamine	ASUTZQLVASHGKV-JDFRZJQESA-N	357-70-0	5.34
adenosine	OIRDTQYFTABQOQ-UHFFFAOYSA-N	58-61-7	5.24
Vidarabine	OIRDTQYFTABQOQ-UHTZMRCNSA-N	5536-17-4	5.24
betaxolol	NWIUTZDMDHAVTP-UHFFFAOYSA-N	63659-18-7	5.20
Morphine,dihydro	IJVCSMSMFSCRME-KBQPJGBKSA-N	509-60-4	5.10
tranylcypromine	AELCINSCMGFISI-DTWKUNHWSA-N	13492-01-8	5.09
tranylcypromine	AELCINSCMGFISI-YGPZHTELSA-N	155-09-9	5.09
